# The practice of evaluating epidemic response in humanitarian and low-income settings: a systematic review

**DOI:** 10.1186/s12916-020-01767-8

**Published:** 2020-11-03

**Authors:** Abdihamid Warsame, Jillian Murray, Amy Gimma, Francesco Checchi

**Affiliations:** Faculty of Epidemiology and Population Health, The London School of Hygiene & Tropical Medicine, London, UK

**Keywords:** Epidemic, Evaluation, Outbreak, Humanitarian, Low-income

## Abstract

**Background:**

Epidemics of infectious disease occur frequently in low-income and humanitarian settings and pose a serious threat to populations. However, relatively little is known about responses to these epidemics. Robust evaluations can generate evidence on response efforts and inform future improvements. This systematic review aimed to (i) identify epidemics reported in low-income and crisis settings, (ii) determine the frequency with which evaluations of responses to these epidemics were conducted, (iii) describe the main typologies of evaluations undertaken and (iv) identify key gaps and strengths of recent evaluation practice.

**Methods:**

Reported epidemics were extracted from the following sources: World Health Organization Disease Outbreak News (WHO DON), UNICEF Cholera platform, Reliefweb, PROMED and Global Incidence Map. A systematic review for evaluation reports was conducted using the MEDLINE, EMBASE, Global Health, Web of Science, WPRIM, Reliefweb, PDQ Evidence and CINAHL Plus databases, complemented by grey literature searches using Google and Google Scholar. Evaluation records were quality-scored and linked to epidemics based on time and place. The time period for the review was 2010–2019.

**Results:**

A total of 429 epidemics were identified, primarily in sub-Saharan Africa, the Middle East and Central Asia. A total of 15,424 potential evaluations records were screened, 699 assessed for eligibility and 132 included for narrative synthesis. Only one tenth of epidemics had a corresponding response evaluation. Overall, there was wide variability in the quality, content as well as in the disease coverage of evaluation reports.

**Conclusion:**

The current state of evaluations of responses to these epidemics reveals large gaps in coverage and quality and bears important implications for health equity and accountability to affected populations. The limited availability of epidemic response evaluations prevents improvements to future public health response. The diversity of emphasis and methods of available evaluations limits comparison across responses and time. In order to improve future response and save lives, there is a pressing need to develop a standardized and practical approach as well as governance arrangements to ensure the systematic conduct of epidemic response evaluations in low-income and crisis settings.

## Background

Infectious disease epidemics continue to pose a substantial risk globally [1]. Epidemics routinely occur in low-income and humanitarian settings [2]. Populations in these settings often do not have the resources to effectively respond to epidemics [3] and as a result are at higher risk of increased morbidity and mortality [4]. Globally, more than 700 million people live in low-income countries [5], while 2 billion live in fragile or conflict-affected settings [6]. Responses to large-scale epidemics or epidemics of newly emergent pathogens tend to generate global attention and corresponding responses incur scrutiny [7–9]. However, evidence on responses to smaller-scale epidemics or epidemics involving well-known pathogens (e.g. measles, cholera) for which effective control measures exist is thought to be limited [10]. Evidence from some limited contexts points to weaknesses in responses ranging from detection, investigation to effective and timely response [11, 12]. However, the practice of epidemic response evaluation has not been systematically assessed in low-income and humanitarian settings. Within public health programming, effective evaluations generate critical evidence and allow for systematic understanding, improvement and accountability of health action [13]. We sought to review the extent to which evaluations of epidemic responses are actually conducted in low-income and crisis settings and describe key patterns in evaluation practice. Specifically, we aimed to (i) identify epidemics reported in low-income and crisis settings, by aetiologic agent, over a recent period; (ii) determine the frequency with which evaluations of responses to these epidemics were conducted; (iii) describe the main typologies of evaluations undertaken; and (iv) identify key gaps and strengths of recent evaluation practice, so as to formulate recommendations.

## Methods

### Scope of the review

This review (PROSPERO registration CRD42019150693) focuses on recent epidemics in low-income settings, defined using the 2018 World Bank criteria [14], as well as epidemics occurring in settings with ongoing humanitarian responses, as reported in the United Nations Office for the Coordination of Humanitarian Affairs’ annual Global Humanitarian Overview. Our search focused on epidemic-prone pathogens commonly occurring in low resource or humanitarian settings and which presented an immediate threat to life. For this reason, our search excluded HIV [15], tuberculosis [16] and Zika [17]. Epidemics occurring within healthcare settings only or within animal populations were considered outside the scope of this review. In order to capture recent trends and assess contemporary reports, we focused on the period 2010–2019.

### Epidemics

#### Search strategy

The following sources were reviewed in order to compile a list of reported epidemics: World Health Organization Disease Outbreak News (WHO DON) [18], UNICEF Cholera platform [19], Reliefweb [20], PROMED [21] and Global Incidence Map [22]. In line with WHO guidance on infectious disease control in emergencies [23], one suspected case of the following was considered to be an epidemic: acute haemorrhagic fevers (Ebola, Lassa fever, Rift valley fever, Crimean-Congo haemorrhagic fever), anthrax, cholera, measles, typhus, plague and polio. For the remainder of the pathogens, we defined an epidemic as an unusual increase in incidence relative to a previously established baseline in a given setting.

We reviewed WHO DON narrative reports to extract metadata on location (country), year, month and pathogen. Reliefweb was searched for reported epidemics using the search engine and the disaster type filter. For the PROMED database, only epidemics rated as 3 or higher in the 5-point rating system (which reflected a higher degree of certainty in the scale of the epidemic and its potential severity) and in which incident cases and deaths were reported were considered for inclusion. The Global Incident Map database was searched utilizing the inbuilt search function filtering results that were out of scope (wrong location, pathogen, etc.) at the source.

We collated all epidemic records into a single database and removed duplicate reports of the same epidemic based on first date and location of occurrence; duplicated included multiple reports within any given database (e.g. an update on an earlier reported epidemic) and reports of the same epidemic in multiple databases. As phylogenetic or spatio-temporal reconstructions of epidemics were mostly unavailable, we assumed that reports of the same pathogen from within the same country and 4-month period referred to the same single epidemic. We decided to split cross-border epidemics (e.g. the West Africa 2013–2016 Ebola epidemic) into one separate epidemic for each country affected, recognizing that responses would have differed considerably across these countries.

#### Screening and data extraction

We compiled epidemic reports from various sources into one database. For each epidemic, information on location (country), year, month and pathogen was extracted using a standardized form (see Additional file 1). For reach evaluation record, information was extracted on a number of variables including type of evaluation, location (country), year, month and pathogen using a standardized form (see Additional file 2).

### Evaluations

#### Search strategy

To determine the availability and quality of epidemic response evaluations within recent epidemics, we undertook a systematic review using PRISMA criteria including peer-reviewed and grey literature. We identified peer-reviewed reports by consulting the MEDLINE, EMBASE, Global Health, Web of Science, Western Pacific Region Index Medicus, PDQ Evidence and Cumulative Index to Nursing and Allied Health Literature (CINAHL) Plus databases. We utilized Google, Google Scholar and Reliefweb, to undertake a comprehensive search of the grey literature. Given previously described challenges in using such search engines [24], we reviewed results from the first 150 hits only. We searched the webpages of major humanitarian and health organizations including the World Health Organization (WHO), United Nations Children’s Fund (UNICEF), Save the Children, International Federation of Red Cross and Red Crescent Societies (IFRC) and Médecins Sans Frontières (MSF) for evaluation records and contacted these organizations to source non-public evaluations identified through this webpage search. Overarching conceptual search terms synonymous with outbreaks, evaluations and humanitarian crises were utilized. The full search strategy can be found in Additional file 3.

We cross-referenced the reported epidemics with the evaluation reports, matching on date (month and year) and location.

#### Inclusion criteria

We limited our search to any record that met the following criteria: any document published in the period 2010–2019 in the English and French languages that examined epidemics within low-income countries and humanitarian settings, as defined above. There were no restrictions on study design.

We excluded records relying exclusively on mathematical models of potential responses as the review was focused on responses that were operationally implemented. We also excluded evaluations of a novel diagnostic or treatment; evaluations that focussed on preparedness, resilience or recovery from an epidemic, as opposed to the epidemic period itself; records addressing other health issues (e.g. reproductive health) in the context of an epidemic; epidemiological studies of the epidemic (e.g. transmission patterns, risk factors) that did not explore the response; records classified as clinical research, opinion or news pieces; and abstracts for which full records could not be accessed.

In assessing the eligibility of records for narrative synthesis, we used a broad definition of epidemic evaluation as one in which:

I. An epidemic was reported to have occurred
II. The intervention(s) being evaluated began after the start of the epidemic and were specifically implemented in response to the epidemic
III. The intervention(s) were assessed on at least one specified criterion (i.e. the report was not merely a description of activities).

#### Screening and data extraction

After removing duplicates, two reviewers independently assessed the relevance of all titles and abstracts based on the inclusion criteria. We retrieved the full text of each article initially meeting the criteria. Two researchers then independently confirmed that full-text records met inclusion criteria. Any disagreements were resolved through discussion and consensus with a third reviewer.

We used the following definitions to classify the type of evaluations retrieved in this search:

- *Formative evaluation*: Evaluation which assesses whether a program or program activity is feasible, appropriate and acceptable before it is fully implemented
- *Process evaluation*: Evaluation which determines whether program activities have been implemented as intended
- *Output evaluation*: Evaluation which assesses progress in short-term outputs resulting from program implementation
- *Outcome/performance evaluation*: Evaluation which assesses program effects in the target population by measuring the progress in the outcomes or outcome objectives that the program is meant to achieve
- *Impact evaluation*: An evaluation that considers ‘positive and negative, primary and secondary long-term effects produced by a development intervention, directly or indirectly, intended or unintended.’ [25]

### Analysis

We undertook a narrative synthesis of the findings and tabulated key characteristics of evaluations. We created an evaluation quality checklist derived from existing standards to grade the quality of the evaluation records. Reference standards included the United Nations’ Evaluation Group (UNEG) Quality Checklist [26], the European Commission Quality Assessment for Final Evaluation Reports [27] and the UNICEF-Adapted UNEG Quality Checklist [28]. We derived 13 evaluation criteria grouped into 4 equally weighted categories: scope, methodology, findings and recommendations. The checklist can be found in Additional file 3.

### Role of the funding source

The funder of the study had no role in study design, data collection, data analysis, data interpretation or writing of the report. The corresponding author had full access to all the data in the study and had final responsibility for the decision to submit for publication.

## Results

### Epidemics

A total of 429 epidemics were identified across 40 low-income and crisis affected countries during the study period (Table 1). The most common pathogens reported were *Vibrio cholerae*, measles, poliovirus and Lassa virus. Epidemics were reported primarily in sub-Saharan Africa, the Middle East and Central Asia. Generally, the more populous countries in each region experienced the highest number of epidemics including Nigeria and the Democratic Republic of Congo in the AFRO region and Pakistan and Sudan in the EMRO region.

**Table 1:** Number of epidemics by outbreak pathogen and World Health Organization regional office

Disease	Number of epidemics by WHO regional office					
	AFRO^a^	EMRO^b^	EURO^c^	PAHO^d^	SEARO^e^	Total
Anthrax	7	4	2	1	0	**14**
Brucellosis	0	1	0	0	0	**1**
CCHF	2	5	1	0	0	**8**
Chikungunya	2	2	0	1	0	**5**
Cholera	145	36	3	10	14	**208**
Dengue	6	11	1	2	11	**31**
Diphtheria	0	1	0	2	0	**3**
Ebola	16	1	0	0	1	**18**
Hepatitis E	2	0	0	0	0	**2**
Japanese Encephalitis	0	1	0	0	4	**5**
Lassa Fever	17	0	0	0	0	**17**
Leishmaniasis	3	0	0	0	0	**3**
Malaria	10	2	0	1	0	**13**
Marburg	1	0	0	0	0	**1**
Measles	20	6	0	1	0	**27**
Meningitis	8	1	0	0	0	**9**
Meningococcal disease	11	1	0	0	0	**12**
Plague	7	0	0	0	0	**7**
Polio	12	11	2	0	1	**26**
Rift Valley Fever	3	2	0	0	0	**5**
Typhoid	0	1	0	0	0	**1**
Yellow fever	11	2	0	0	0	**13**
Grand Total	**283**	**88**	**9**	**18**	**31**	**429**

^a^WHO African Regional Office

^b^WHO Eastern and Mediterranean Regional Office

^c^WHO European Regional Office

^d^WHO Pan American Regional Office

^e^WHO South East Asia Regional Office

### Evaluations

**Fig. 1:**
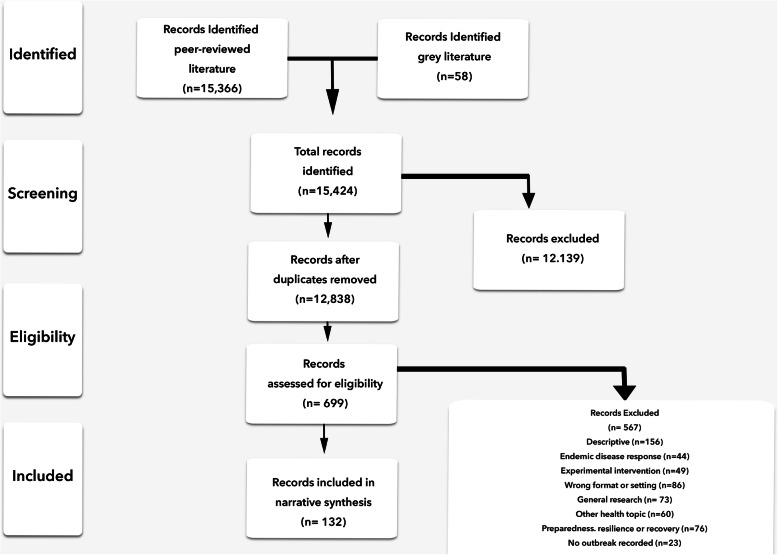
Evaluation records screened

A total of 15,124 records were identified and screened based on title and abstract (Fig. 1). The full text of 699 records was assessed for eligibility. A final tally of 132 records was carried forward for cross referencing against reported epidemics and for narrative synthesis [29–160]. See Additional file 2 for full list of included evaluations.

#### Evaluation characteristics

**Fig. 2:**
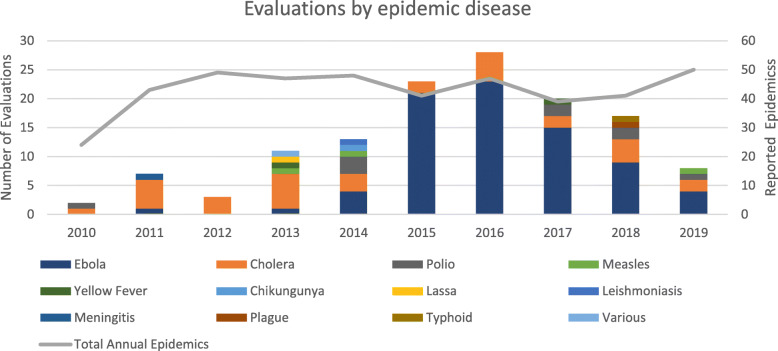
Annual availability of response evaluation by epidemic disease compared to total annual epidemics

More than half of the evaluation records assessed outcome of the response, with a substantial number of process and output evaluations (Table 2). Very few evaluations could be classified as impact or formative evaluations while 4 studies were considered to be of mixed typology. Half of the evaluations reported (*n* = 66) utilizing a mix of primary and secondary data while approximately a quarter of evaluations utilized either mainly primary (*n* = 36) or secondary data (*n* = 30). Additionally, more than half of evaluations (*n* = 78) collected a mixture of quantitative and qualitative data while a smaller proportion relied on either qualitative data (*n* = 18) and quantitative data (*n* = 37). Few records (*n* = 9) had no explicit evaluation framework or criteria while the majority (*n* = 123) did refer to some evaluation criteria including the OECD evaluation criteria. However, only few (*n* = 10) presented an explicitly named framework which anchored the evaluation approach. Effectiveness was the most widely used evaluation criterion while the most widely evaluated activities included coordination, vaccination, contact tracing, case management and community sensitization. See Additional file 2 for full results. There was an improvement in the availability of evaluation reports over time with fewer evaluations in the first 3 years of the decade (*n* = 10) compared to the last 3 years (*n* = 43) (Fig. 2). Lastly, where evaluations were published, there was an average of 2 years between the onset of an epidemic and publication of the response evaluation.

**Table 2:** Distribution of response evaluations by type

Type of evaluation	Number of evaluations
Formative	4 (3.0%)
Process	27 (20.5%)
Output	14 (10.6%)
Outcome	74 (56.0%)
Impact	9 (6.8%)
Mixed	4 (3.0%)
Total	132 (100%)

#### Quality findings

Quality scores of evaluation reports ranged from 31 to 96 on a 100-point scale. The average quality score of evaluations in low-income settings was 76 compared to 68 in middle-income countries. The average quality scores of evaluations undertaken in humanitarian versus non-humanitarian settings did not differ substantially (76.7 vs 75.2) nor between mid-epidemic versus post epidemic (74.4 vs 76.9). Quality scores ranged amongst disease pathogens with the highest average quality scores for evaluation of measles epidemics (88.4) and the lowest for evaluations of leishmaniosis epidemics (57.6). Additionally, there appeared to be an improvement in the quality of evaluation reports over time with reports in the first 3 years of the decade averaging a score of 64 compared to a score of 80 in the last 3 years. Although the majority of evaluations (*n* = 104) did identify and utilize existing information and documentation, few (*n* = 28) provided an appraisal of quality or reliability of these data sources. For the most part, evaluation studies did score well in presenting the rationale of the evaluation (average score = 0.88), providing the contextual information (average score 0.92) and clarifying the evaluation timeline (average score 0.82). They scored less well in providing sufficient detail on the methodological approach suitable to the scope (average score 0.77) as well as detailing limitations of the evaluation (average score 0.61).

#### Evaluation coverage

We were able to link approximately 9% (*n* = 39) of epidemics with one or more response evaluations (Table 3). Some evaluation reports (*n* = 18) covered responses in multiple countries. A large number of evaluations focused on the same epidemic; for example, 47 evaluations were undertaken to assess the West Africa Ebola epidemic (2013–2016). There were approximately equal numbers of post-epidemic (56%) and mid-epidemic evaluations (44%). The majority of epidemic response evaluations (87%) were undertaken in countries which had experienced humanitarian emergencies during the study period. Furthermore, 83% of response evaluations were undertaken in the WHO Africa region, 8% in the Eastern Mediterranean region and the remainder in the Americas region. Two evaluations could not be linked to an epidemic as the epidemic occurred outside of the study period (prior to 2010).

**Table 3:** Number of epidemic evaluations by pathogen, country, year and whether the evaluation was performed during or after the epidemic

Disease	Country	Year	Evaluations	During epidemic	Post epidemic	Average quality score	Average publication delay (years)
Cholera	Benin	2012	1	0	1	42.3	2.0
Cholera	Chad	2010	1	1	0	69.2	1.0
Cholera	DRC	2012	1	1	0	61.5	2.0
Cholera	Guinea	2012	1	0	1	73.1	1.0
Cholera	Haiti	2010	8	6	2	77.4	2.8
Cholera	Haiti	2012	2	2	0	92.3	3.5
Cholera	Haiti	2013	1	0	1	88.5	4.0
Cholera	Malawi	2015	2	1	1	88.5	1.0
Cholera	Nigeria	2010	1	1	0	69.2	2.0
Cholera	Nigeria	2015	1	0	1	96.2	0.0
Cholera	Sierra Leone	2012	2	1	1	76.9	1.0
Cholera	Somalia	2016	1	1	0	84.6	2.0
Cholera	Somalia	2017	1	0	1	92.3	2.0
Cholera	South Sudan	2014	1	1	0	92.3	0.0
Cholera	South Sudan	2015	1	1	0	96.2	1.0
Cholera	Uganda	2015	1	0	1	46.2	1.0
Cholera	Yemen	2016	3	2	1	76.9	2.3
Cholera	Yemen	2017	2	0	2	96.2	1.0
Ebola	DRC	2012	1	0	1	73.1	5.0
Ebola	DRC	2018	1	1	0	88.5	1.0
Ebola	Guinea	2014	23	10	13	76.7	2.6
Ebola	Liberia	2014	36	13	23	73.2	2.2
Ebola	Mali	2014	1	0	1	34.6	2.0
Ebola	Nigeria	2014	11	5	6	51.3	1.4
Ebola	Sierra Leone	2014	47	24	23	77.2	2.1
Ebola	Uganda	2012	3	0	3	69.2	1.0
Ebola	Uganda	2012	2	0	2	53.9	2.0
Lassa Fever	Nigeria	2012	1	0	1	65.4	1.0
Measles	Ethiopia	2011	1	0	1	92.3	3.0
Measles	Madagascar	2018	1	0	1	92.3	1.0
Plague	Madagascar	2017	1	0	1	65.4	1.0
Polio	Chad	2011	1	0	1	84.6	3.0
Polio	Ethiopia	2013	1	0	1	88.5	5.0
Polio	Nigeria	2010	1	1	0	92.3	4.0
Polio	Nigeria	2018	1	1	0	65.4	1.0
Polio	Somalia	2013	2	0	2	65.4	2.5
Polio	Ukraine	2015	1	0	1	80.8	2.0
Yellow fever	DRC	2016	1	0	1	84.6	1.0
Yellow fever	Uganda	2010	1	0	1	84.6	3.0

**Fig. 3:**
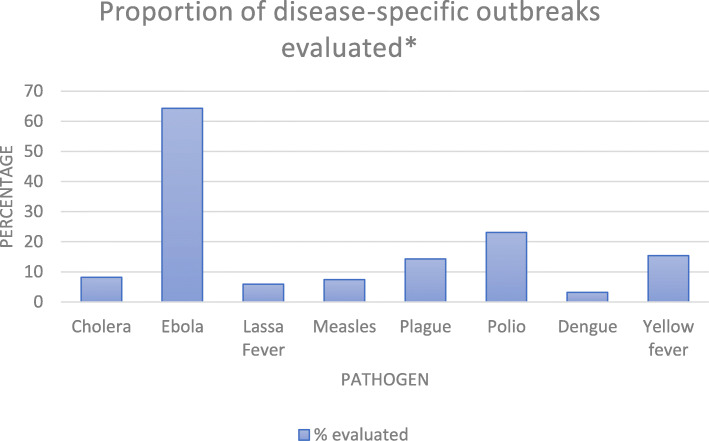
Proportion of outbreaks evaluated for each disease. Asterisk indicates no evaluation reports were found for outbreaks of anthrax, brucellosis, diphtheria, hepatitis E, Japanese encephalitis, malaria, Marburg haemorrhagic fever, Meningococcal disease and Rift Valley fever

Coverage of response evaluations varied by disease (Fig. 3). Ebola epidemics had the highest coverage of response evaluations with 64% of reported epidemics having a response evaluation with Lassa fever epidemics having the lowest coverage (6%). No response evaluations were found for epidemics of anthrax, brucellosis, diphtheria, hepatitis E, Japanese encephalitis, malaria, Marburg haemorrhagic fever, Meningococcal disease and Rift Valley fever epidemics.

## Discussion

To our knowledge, this is the first study to systematically explore the coverage and characteristics of epidemic evaluations in low resource and humanitarian settings. The low proportion of epidemics with evidence of evaluations in settings with low resources and high needs suggests an inequity [3] requiring urgent addressing. The lack of evaluations also points to a deficit in the accountability to affected populations, a key principle in humanitarian response [161]. Without the availability of rigorous, high quality and standardized response evaluations, affected populations are unable to hold responders to account and have no recourse to redress [29].

The 2-year delay between the onset of an epidemic and the publication of an evaluation report is a barrier to efficient dissemination of response findings. Reducing this delay can potentially be of use in addressing existing delays in global disease response [162]. More importantly, it represents a missed opportunity to enact changes in a timely manner.

There was considerable variability in the criteria considered by the evaluations, including quality, coverage, efficiency, effectiveness, relevance, appropriateness, fidelity or adherence, acceptability and feasibility. Various combinations of these criteria were used to assess a large number of response activities. Within a given epidemic as well as across epidemics, individual evaluations utilized a wide array of differing assessment criteria and assessed response activities. This variability combined with a lack of an overarching evaluation framework makes it difficult to compare evaluations to the same response or generalize their conclusions. This finding is consistent with a previous study on the use of public health evaluation frameworks in emergencies [1] and underscores a need for an approach and corresponding toolset that standardizes the evaluation of epidemics in these settings. We have previously proposed an overarching framework for such a unifying approach [1].

The term ‘impact’ was frequently found in the assessed evaluations, but very few of the assessed evaluations could be classified as impact evaluations, reflecting the relatively high technical and resource barriers required to conduct a robust impact evaluation. Furthermore, there were marked differences in the number of evaluations by disease pathogen. Whereas one would expect well-characterized diseases with frequent epidemics to have the most evaluations, the opposite was largely found. Approximately two thirds of the Ebola epidemics recorded had response evaluations compared to 8% of cholera epidemics and 5% of Lassa fever epidemics. This is despite the annual attributable deaths due to cholera being 120,000 [163] and Lassa fever 5000 [164]. This finding is in line with previous research suggesting that more severe epidemics or epidemics that threaten large numbers of people do not necessarily receive more timely response [162]. The overrepresentation of Ebola response evaluations is perhaps reflective of a number of factors such as the unprecedented scale and better resourcing of the response of the West African outbreak. Superficially, this overrepresentation could perhaps be due to the poor containment efforts at the outset of the epidemic leading to international spread and in turn generating higher international attention and scrutiny. However, the scale-up and securitization of the response and subsequent increased scrutiny may have also reflected the proximity of the epidemic to developed countries [165, 166].

### Implications of this study

The gaps identified in this review are particularly pertinent to future evaluations of the COVID-19 pandemic which has reached most low-income and humanitarian settings [167]. Infectious disease epidemics have been known to exploit and exacerbate social inequalities within societies for some time [168, 169]. This review highlights the current global inequality in the response to these epidemics as gauged by the number of epidemics in low-income settings and the paucity of evaluation reports. This nonexistence or lack of publication of these critical evaluations prevents improvements to future public health response. The lack of uniformity of available evaluations limits comparison of the findings across responses and across time, precluding tracking of whether epidemic responses do in fact improve over time, globally or at regional level. The quality limitations of some of the evaluations hinder the strength of inference and applicability of their findings. There is a need to overcome this limitation in order to enable future research to be conducted on the findings of response valuations. More specifically, future reviews of epidemic response should attempt to synthesize quantitative effects of response interventions and may benefit from SWiM guidelines where appropriate [170].

### Limitations

Our study relied overwhelmingly on publicly available evaluations. It is possible that the disparity between the number of epidemics, their responses and their subsequent evaluations could be overstated as evaluation findings might simply be kept internal and not shared more widely. However, the effect is largely the same as internal evaluations are only of benefit to the commissioning organization and cannot be used more widely. Additionally, we assumed that all epidemics were responded to and therefore should have been evaluated. However, we did not know the true proportion of epidemics that were responded to and therefore could potentially overestimate the gap between evaluations and epidemics. On the other hand, the use of a 4-month decision rule to combine multiple reports of epidemics within the same country could have resulted in an underestimate of the total number of epidemics and thus an overestimate of evaluation coverage. We did not look at records that were not written in English or French and could potentially have missed some evaluations.

## Conclusion

The relative paucity of evaluated epidemics, the disproportionate number of evaluations focusing on a limited number of epidemics together with constrained resource availability in low-income settings suggests the need for a governance arrangement or systematic mechanism that would trigger the conduct of evaluations, no matter what. The need for strengthening global governance mechanisms related to infectious disease epidemics and related challenges have been discussed [171, 172]. We suggest that arrangements should cover the criteria that should trigger an evaluation, the timing of evaluation, the composition and affiliation of the evaluation team, funding, minimum evaluation standards (e.g. a common scope and framework) and publication steps.

Approximately 2 billion people live in conflict-affected or fragile states and are at risk of increased morbidity and mortality due to epidemics every year. Robust epidemic response evaluations seek to improve response through critically assessing the performance of response interventions in a given context. However, evaluations of epidemic response are not a stand-alone activity but rather must be integrated into a cycle of preparedness and recovery in order to reach their full utility [173]. The lessons learned from an evaluation should concretely support all responders to better prepare for similar epidemic and to support health system recovery.

## Supplementary information

**Additional file 1.** List of Epidemics.

**Additional file 2.** Evaluation Extraction Table.

**Additional file 3.** Search Strategy & Evaluation Quality Checklist.

## Data Availability

All data generated or analysed during this study are included in this published article and its supplementary information files.
