# Supplementary material for: The practice of evaluating epidemic response in humanitarian and low-income settings: a systematic review
Source: BMC Med. 2020 Nov 3;18:315. doi: 10.1186/s12916-020-01767-8 (PMC7606030; doi:10.1186/s12916-020-01767-8)
Supplement: Supplementary file 2 — Additional file 2. Evaluation Extraction Table. [file 12916_2020_1767_MOESM2_ESM.docx]

## Extraction table of included studies

| Study ID | Pathogen | Country | Humanitarian setting | Evaluation Type | Timing | Commissioned | Evaluation framework or criteria | Data collection | Indicator Type | Data | Quality Score |
| --- | --- | --- | --- | --- | --- | --- | --- | --- | --- | --- | --- |
| Malik 2014[1] | Chikungunya | Yemen | No | Outcome | mid-outbreak | external | effectiveness | mixed | outcome | mixed | 69.23 |
| [Makoutodé](https://www.cairn.info/publications-de-Michel-Makoutod%C3%A9--60810.htm) 2010[2] | Cholera | Benin | No | Process, | Post outbreak | Internal | Process | mixed | Process | mixed | 42.31 |
| Aka 2013[3] | Cholera | Benin | Yes | Output | post-outbreak | internal | Quality, relevance and accountability, effectiveness and efficiency of management | mixed | output | qualitative | 80.77 |
| ACF 2011[4] | Cholera | Chad | Yes | Process | Mid outbreak | internal | Effectiveness | Primary | Process, Impact | mixed | 69.23 |
| Grayel 2014[5] | Cholera | DRC | Yes | Process | Mid outbreak | internal | Process | Primary | Process | mixed | 61.54 |
| Ciglenecki 2013[6] | Cholera | Guinea | No | Feasibility | post-outbreak | internal | Feasibility, cost, timeliness, and acceptability | secondary | process, outcome | quantitative | 73.08 |
| Cavallaro 2011[7] | Cholera | Guinea-Bissau | No | Outcome | mid-outbreak | external | effectiveness | primary | outcome | quantitative | 88.46 |
| Ciglenecki 2013[8] | Cholera | Haiti | Yes | Outcome | mid-outbreak | internal | effectiveness | primary | outcome | quantitative | 80.77 |
| Routh 2017[9] | Cholera | Haiti | Yes | Outcome | post-outbreak | internal | cost-effectiveness, efficiency | primary | process | quantitative | 88.46 |
| Severe 2016[10] | Cholera | Haiti | Yes | Outcome | mid-outbreak | internal | vaccine effectiveness | mixed | process, outcome | mixed | 88.46 |
| Ivers 2015[11] | Cholera | Haiti | Yes | Process | mid-outbreak | internal | vaccine effectiveness | mixed | process, outcome | mixed | 96.15 |
| Santa-Olalla 2013[12] | Cholera | Haiti | Yes | Output | mid-outbreak | internal | effectiveness | primary | output, outcome | quantitative | 73.08 |
| Tauxe 2011[13] | Cholera | Haiti | Yes | Impact | mid-outbreak | internal | effectiveness | primary | impact | quantitative | 76.92 |
| Dhillon 2012[14] | Cholera | Haiti | Yes | Process | post-outbreak | external | fidelity (quality of reporting0 | secondary | output | quantitative | 92.31 |
| Teng 2014[15] | Cholera | Haiti | Yes | Feasibility | mid-outbreak | internal | efficiency (timeliness), | mixed | process, outcome | quantitative | 88.46 |
| Grayel 2011[16] | Cholera | Haiti | Yes | Process | Mid outbreak | internal | Effectiveness | Primary | Impact | mixed | 61.54 |
| Dobai 2016[17] | Cholera | Haiti , Dominican Republic | Yes | Impact | post-outbreak | internal | relevance, impact, coverage, sustainability and connectedness of the interventions and assess the cooperation, coordination and effectiveness of the accountability process | mixed | output, outcome | mixed | 92.31 |
| Tappero 2011[18] | Cholera | Haiti, Dominican Republic | Yes | Output | mid-outbreak | internal | n/a | secondary | outputs, outcome | mixed | 53.85 |
| Khonje 2012[19] | Cholera | Malawi | No | Outcome | post-outbreak | internal | effectiveness | mixed | outcome | mixed | 53.85 |
| Msyamboza 2016[20] | Cholera | Malawi | No | Feasibility | mid-outbreak | internal | feasibility, acceptability | primary | process, outcome | quantitative | 88.46 |
| Oladale 2012[21] | Cholera | Nigeria | Yes | Process | mid-outbreak | external | n/a | primary | process | mixed | 69.23 |
| Fogden 2015[22] | Cholera | Nigeria | Yes | Outcome | post-outbreak | internal | 1) Relevance and appropriateness, 2) Efficiency, 3) Effectiveness, 4) Coverage, 5) Coherence, and 6) Sustainability and connectedness | mixed | output, outcome | mixed | 96.15 |
| Rees-Gildea 2013[23] | Cholera | Sierra Leone | No | Impact | post-outbreak | internal | Quality, relevance and accountability, effectiveness and efficiency of management | mixed | outcome, impact | mixed | 80.77 |
| OXFAM 2012[24] | Cholera | Sierra Leone | No | Impact | mid-outbreak | internal | Global Humanitarian Indicator Tool. | mixed | outcome, impact | mixed | 73.08 |
| Mcgowan 2018[25] | Cholera | Somalia | Yes | Process | post-outbreak | internal | relevance, appropriateness, effectiveness, accountability, coordination, evaluation and learning, management and staffing, efficiency | mixed | process | mixed | 92.31 |
| Dyson 2018[26] | Cholera | Somalia | Yes | Outcome | mid-outbreak | internal | appropriate in terms of: scale, scope, timeliness, and relevance; standards of quality technical programming; effectiveness , efficiency , connectedness and coherence | mixed | process, outcome | mixed | 84.62 |
| Azman 2016[27] | Cholera | South Sudan | Yes | Process | mid-outbreak | internal | vaccine effectiveness | mixed | process, outcome | mixed | 96.15 |
| Gauthier 2014[28] | Cholera | South Sudan | Yes | Outcome | mid-outbreak | internal | Efficiency, effectiveness, appropriateness | mixed | process, outcome, impact | mixed | 92.31 |
| Bwire 2016[29] | Cholera | Uganda, Malawi | No | Output | post-outbreak | external | n/a | secondary | n/a | quantitative | 46.15 |
| Dureab 2019[30] | Cholera | Yemen | Yes | Process | mid-outbreak | external | Timeliness | secondary | process | quantitative | 69.23 |
| Altmann 2017[31] | Cholera | Yemen | Yes | Outcome | mid-outbreak | internal | effectiveness | primary | output, outcome | mixed | 80.77 |
| Federspiel 2018[32] | Cholera | Yemen | Yes | Process | post-outbreak | external | n/a | secondary | n/a | qualitative | 53.85 |
| Darcy 2018[33] | Cholera | Yemen | Yes | Outcome | post-outbreak | internal | appropriateness., coordination ,effectiveness, efficiency, coverage, connectedness | mixed | output, process. Outcome | mixed | 96.15 |
| Spiegel 2019[34] | Cholera | Yemen | Yes | Process | post-outbreak | external | GTFCC 3 approaches | mixed | outcome, impact | mixed | 80.77 |
| Ashbaugh 2017[35] | Ebola | DRC | No | Outcome | post-outbreak | external | effectiveness | secondary | output | quantitative | 73.08 |
| McGowan 2019[36] | Ebola | DRC | Yes | Process | mid-outbreak | internal | Core Humanitarian Standard on Quality and Accountability | mixed | process | mixed | 88.46 |
| Hennessee 2018[37] | Ebola | Guinea | No | Process | mid-outbreak | external | effectiveness | mixed | process | mixed | 88.46 |
| Diallo 2015[38] | Ebola | Guinea | No | Outcome | mid-outbreak | external | effectiveness based on SBM-R performance standards for IPC | mixed | outcome | mixed | 61.54 |
| Fu 2015[39] | Ebola | Guinea | No | Outcome | mid-outbreak | external | effectiveness | primary | outcome | qualitative | 69.23 |
| Standley 2019[40] | Ebola | Guinea | No | Outcome | mid-outbreak | internal | timeliness, adherence (fidelity) | primary | output, process | mixed | 69.23 |
| Lee 2016[41] | Ebola | Guinea | No | Output | mid-outbreak | external | effectiveness (sensitivity) | secondary | output | quantitative | 80.77 |
| Requesa 2017[42] | Ebola | Guinea | No | Process | mid-outbreak | external | fidelity | mixed | input, output. Process, outcome | mixed | 88.46 |
| Soeters 2018[43] | Ebola | Guinea | No | Outcome | mid-outbreak | internal | effectiveness | primary | outcome | mixed | 73.08 |
| Kokki 2017[44] | Ebola | Guinea | No | Outcome | post-outbreak | internal | preparation, appropriateness and implementation of ECDC plans, procedures, structures • relevance, appropriateness, efficiency and effectiveness of the field activities • impact of this deployment on ECDC work programme. | mixed | process, outcome | qualitative | 88.46 |
| Fitzpatrick 2017[45] | Ebola | Guinea, Liberia, Sierra Leone | No | Outcome | post-outbreak | internal | operational research domains | secondary | output, outcome | qualitative | 57.69 |
| Murray 2015[46] | Ebola | Guinea, Liberia, Sierra Leone | No | Outcome | mid-outbreak | internal | Relevance and appropriateness, Efficiency, Effectiveness connectedness | mixed | process, outcome | qualitative | 84.62 |
| UNICEF 2017[47] | Ebola | Guinea, Liberia, Sierra Leone | No | Outcome | post-outbreak | internal | effectiveness, efficiency, internal coordination, external coordination and accountability | mixed | output | mixed | 96.15 |
| WHO 2015[48] | Ebola | Guinea, Liberia, Sierra Leone | No | Process | mid-outbreak | external | timeliness, appropriateness, scale, effectiveness), including (i) coordination within the Organization and with Member States (ii) resource mobilization and (iii) communications; | mixed | n/a | mixed | 73.08 |
| Shepherd 2017[49] | Ebola | Guinea, Liberia, Sierra Leone | No | Outcome | post-outbreak | internal | relevance, coherence and appropriateness; coverage; coordination and connectedness; effectiveness; | mixed | output | mixed | 88.46 |
| Sadaphal 2017[50] | Ebola | Guinea, Liberia, Sierra Leone | No | Outcome | post-outbreak | internal | overall effectiveness, effectiveness of different programmatic components, | mixed | output, outcome | mixed | 92.31 |
| Sadaphal 2018[51] | Ebola | Guinea, Liberia, Sierra Leone | No | Outcome | post-outbreak | internal | overall effectiveness, effectiveness of different programmatic components, | mixed | output, outcome | mixed | 92.31 |
| Sadaphal 2018[52] | Ebola | Guinea, Liberia, Sierra Leone | No | Outcome | post-outbreak | internal | overall effectiveness, effectiveness of different programmatic components, | mixed | output, outcome | mixed | 92.31 |
| HoC 2016[53] | Ebola | Guinea, Liberia, Sierra Leone | No | Outcome | post-outbreak | external | timeliness, resource allocation | mixed | n/a | qualitative | 65.38 |
| Lupel 2017[54] | Ebola | Guinea, Liberia, Sierra Leone | No | Outcome | post-outbreak | internal | leadership and operational direction; scaling up the response; coordination; strategic communications and community engagement; raising the profile of the response. | mixed | n/a | mixed | 61.54 |
| Bell 2016[55] | Ebola | Guinea, Liberia, Sierra Leone, Mali, Nigeria | No | Output | post-outbreak | internal | n/a | secondary | output | mixed | 34.62 |
| Summers 2014[56] | Ebola | Liberia | No | Process | mid-outbreak | internal | county specific response plans | primary | n/a | qualitative | 57.69 |
| Swanson 2018[57] | Ebola | Liberia | No | Outcome | post-outbreak | external | effectiveness | secondary | output, outcome | quantitative | 73.46 |
| Lindblade 2015[58] | Ebola | Liberia | No | Impact | mid-outbreak | internal | effectiveness | secondary | outcome, impact | quantitative | 80.77 |
| Cardile 2016[59] | Ebola | Liberia | No | Outcome | post-outbreak | internal | effectiveness | primary | outcome | mixed | 61.54 |
| De wit 2016[60] | Ebola | Liberia | No | Output | mid-outbreak | internal | fidelity | secondary | output | mixed | 42.31 |
| Wolfe 2017[61] | Ebola | Liberia | No | Outcome | mid-outbreak | internal | effectiveness | primary | output, outcome | mixed | 88.46 |
| Katawera 2019[62] | Ebola | Liberia | No | Output | mid-outbreak | internal | efficiency, effectiveness | secondary | output, outcome | quantitative | 73.08 |
| Kouaidio 2015[63] | Ebola | Liberia | No | Outcome | mid-outbreak | internal | effectiveness | primary | output, outcome | quantitative | 65.38 |
| Sepers 2019[64] | Ebola | Liberia | No | Outcome | mid-outbreak | internal | effectiveness | mixed | output, outcome | mixed | 88.46 |
| Oji 2018[65] | Ebola | Liberia | No | Outcome | mid-outbreak | internal | effectiveness | primary | process, outcome | mixed | 96.15 |
| Nyenswah 2016[66] | Ebola | Liberia | No | Outcome | post-outbreak | internal | crisis leadership tasks framework | secondary | outcome | qualitative | 84.62 |
| Munodawafa 2018[67] | Ebola | Liberia | No | Outcome | post-outbreak | internal | effectiveness | mixed | outcome | mixed | 88.46 |
| Nevin 2016[68] | Ebola | Liberia | No | Process | post-outbreak | internal | effectiveness, timeliness | mixed | output | mixed | 65.38 |
| Abramowitz 2015[69] | Ebola | Liberia | No | Process, Outcome | mid-outbreak | internal | effectiveness | mixed | outcome | mixed | 80.77 |
| YMCA 2015[70] | Ebola | Liberia | No | Outcome | mid-outbreak | internal | Relevance, Equity, Impact & Effectiveness, Efficiency | mixed | n/a | qualitative | 65.38 |
| GC 2015[71] | Ebola | Liberia | No | Output | post-outbreak | internal | n/a | mixed | output, outcome | mixed | 55.38 |
| Carafano 2015[72] | Ebola | Liberia | No | Outcome | post-outbreak | external | n/a | secondary | n/a | qualitative | 30.77 |
| Brown 2014[73] | Ebola | Liberia, Sierra Leone | No | Outcome | mid-outbreak | internal | WHO guidelines | primary | output, outcome | quantitative | 73.09 |
| Hurtado 2018[74] | Ebola | Liberia, Sierra Leone, Guinea | No | Output | post-outbreak | external | availability | secondary | output | quantitative | 84.62 |
| Elemuwa 2015[75] | Ebola | Nigeria | Yes | Outcome | post-outbreak | internal | effectiveness | secondary | outcome | qualitative | 53.85 |
| Oleribe 2015[76] | Ebola | Nigeria | Yes | Outcome | post-outbreak | external | effectiveness | secondary | outcome | qualitative | 38.46 |
| Asuzu 2015[77] | Ebola | Nigeria | Yes | Outcome | post-outbreak | internal | effectiveness | secondary | processes, outcomes | mixed | 46.15 |
| Vaz 2016[78] | Ebola | Nigeria | Yes | Outcome | post-outbreak | internal | effectiveness | secondary | output, outcome | mixed | 50.00 |
| Li 2016[79] | Ebola | Sierra Leone | No | Process | mid-outbreak | internal | impact | mixed | output, outcome | quantitative | 69.23 |
| Ratnayake 2016[80] | Ebola | Sierra Leone | No | Process | mid-outbreak | internal | CEBS SOP | primary | process | mixed | 88.46 |
| Youkee 2015[81] | Ebola | Sierra Leone | No | Outcome | mid-outbreak | internal | effectiveness | primary | outcome | quantitative | 92.31 |
| Vogt 2015[82] | Ebola | Sierra Leone | No | Outcome | mid-outbreak | internal | effectiveness | primary | outcome | quantitative | 88.46 |
| Logue 2017[83] | Ebola | Sierra Leone | No | Outcome | mid-outbreak | internal | effectiveness | primary | output, outcome | quantitative | 61.54 |
| Stone 2016[84] | Ebola | Sierra Leone | No | Process | mid-outbreak | internal | CEBS SOP | primary | process | mixed | 88.46 |
| Olu 2016[85] | Ebola | Sierra Leone | No | Outcome | post-outbreak | external | effectiveness | mixed | output, outcome | mixed | 96.15 |
| Senga 2017[86] | Ebola | Sierra Leone | No | Outcome | post-outbreak | internal | effectiveness | primary | output, outcome | quantitative | 84.62 |
| Oza 2017[87] | Ebola | Sierra Leone | No | Output | mid-outbreak | internal | reliability | primary | output | mixed | 76.92 |
| Jobanputra 2016[88] | Ebola | Sierra Leone | No | Outcome | mid-outbreak | internal | effectiveness | mixed | outcome | mixed | 80.77 |
| Gleason 2015[89] | Ebola | Sierra Leone | No | Output | mid-outbreak | internal | timeliness | primary | process | quantitative | 80.77 |
| Jia 2015[90] | Ebola | Sierra Leone | No | Outcome | mid-outbreak | external | effectiveness, efficiency | secondary | output, outcome | quantitative | 65.38 |
| Ilesanmi 2016[91] | Ebola | Sierra Leone | No | Process | post-outbreak | external | CDC updated guidelines for evaluating public health surveillance systems - simplicity, stability, acceptability, flexibility, representativeness, sensitivity, positive predictive value and data quality | mixed | process, outcome | mixed | 96.15 |
| Garde 2016[92] | Ebola | Sierra Leone | No | Outcome | mid-outbreak | internal | effectiveness | primary | output, outcome | quantitative | 76.92 |
| Nielson 2015[93] | Ebola | Sierra Leone | No | Process | mid-outbreak | internal | fidelity | primary | process, outcome | mixed | 96.15 |
| Lochlainn 2018 [94] | Ebola | Sierra Leone | No | Feasibility | mid-outbreak | internal | effectiveness | primary | output, outcome | mixed | 84.62 |
| Jones-Konneh 2018[95] | Ebola | Sierra Leone | No | Outcome | mid-outbreak | internal | effectiveness | mixed | outcome | mixed | 34.62 |
| Hermans 2017[96] | Ebola | Sierra Leone | No | Outcome | post-outbreak | internal | effectiveness | secondary | output, outcome | quantitative | 84.62 |
| Cancedda 2016[97] | Ebola | Sierra Leone | No | Outcome | mid-outbreak | internal | effectiveness | secondary | output, outcome | quantitative | 46.15 |
| Lokuge 2016[98] | Ebola | Sierra Leone | No | Output | mid-outbreak | internal | effectiveness | secondary | output, outcome | quantitative | 88.46 |
| Stehling-Ariza 2016[99] | Ebola | Sierra Leone | No | Impact | post-outbreak | external | impact | secondary | outcome | mixed | 80.77 |
| Miller 2015[100] | Ebola | Sierra Leone | No | Outcome | mid-outbreak | internal | effectiveness | primary | output, outcome | quantitative | 53.85 |
| Fearon 2017[101] | Ebola | Sierra Leone | No | Output | post-outbreak | internal | HIT evaluation methodology | mixed | output, process. Outcome | mixed | 80.77 |
| YMCA 2016[102] | Ebola | Sierra Leone | No | Outcome | mid-outbreak | internal | effectiveness and impact, relevance, equity, impact, efficiency | primary | output | mixed | 65.38 |
| Cascioli 2014[103] | Ebola | Sierra Leone | No | Outcome | mid-outbreak | internal | quality and effectiveness | mixed | n/a | mixed | 53.85 |
| Platt 2016[104] | Ebola | Sierra Leone | No | Outcome | post-outbreak | internal | OECD/DAC criteria | mixed | outcome | mixed | 92.31 |
| Bayntun 2016[105] | Ebola | Sierra Leone | No | Process | post-outbreak | internal | n/a | mixed | n/a | qualitative | 61.54 |
| TKG 2016[106] | Ebola | Sierra Leone | No | Outcome | post-outbreak | internal | efficiency, effectiveness, accountability, adherence to standards | mixed | output | mixed | 92.31 |
| Batilo Momoh 2016[107] | Ebola | Sierra Leone | No | Outcome | post-outbreak | internal | relevance, effectiveness, efficiency, sustainability, impact | mixed | output, outcome | mixed | 88.46 |
| Age Interntional 2015[108] | Ebola | Sierra Leone | No | Impact | mid-outbreak | internal | relevance, effectiveness, efficiency, impact, accessibility, partnership | mixed | output | mixed | 96.15 |
| Adams 2015[109] | Ebola | Sierra Leone, Liberia | No | Impact | mid-outbreak | internal | Effectiveness and impact, Adherence to global standards, Accountability to beneficiaries, Partnerships, Application and generation of learning | mixed | output | mixed | 96.15 |
| Borchert 2011[110] | Ebola | Uganda | No | Outcome | post-outbreak | internal | effectiveness | secondary | outcome | mixed | 73.08 |
| Mbonye 2014[111] | Ebola | Uganda | No | Outcome | post-outbreak | internal | effectiveness | secondary | outcome | mixed | 53.85 |
| Thormar 2013[112] | Ebola | Uganda | No | Outcome | post-outbreak | internal | Relevance, efficiency | mixed | outcome | qualitative | 69.23 |
| Abubakar 2014[113] | Leishmaniosis | South Sudan | Yes | Outcome | post-outbreak | internal | effectiveness | secondary | output, outcome | quantitative | 57.69 |
| Wallace 2014[114] | Measles | Ethiopia | No | Process | post-outbreak | external | cost-effectiveness, efficiency | mixed | process, output | mixed | 92.31 |
| Lowe 2019[115] | Measles | Madagascar | Yes | Process | post-outbreak | internal | relevance, appropriateness, technical quality, effectiveness, efficiency, | mixed | process | mixed | 92.31 |
| Ntshoe 2013[116] | Measles | South Sudan | No | Impact | post-outbreak | internal | impact | primary | output, impact | quantitative | 80.77 |
| Yamageo 2011[117] | Meningitis | Burkina Faso | No | Process | Post outbreak | Unknown | Process | mixed | Process | mixed | 73.08 |
| Heitzinger 2018[118] | Plague | Madagascar | No | Process | post-outbreak | internal | fidelity (operational research domains) | mixed | outputs, outcome | mixed | 65.38 |
| Ndiaye 2016[119] | Polio | Chad | Yes | Outcome | post-outbreak | internal | effectiveness | mixed | output, outcome | quantitative | 84.62 |
| Tegegne 2018[120] | Polio | Ethiopia | No | Outcome | post-outbreak | internal | effectiveness | mixed | output, outcome | mixed | 88.46 |
| Gammino 2014[121] | Polio | Nigeria | Yes | Process | mid-outbreak | external | SIA guidelines | primary | process | mixed | 92.31 |
| Nkwogu 2018[122] | Polio | Nigeria | Yes | Outcome | mid-outbreak | internal | effectiveness | mixed | output, outcome | mixed | 88.46 |
| Adamu 2019[123] | Polio | Nigeria | Yes | Outcome | mid-outbreak | internal | effectiveness | mixed | output, outcome | quantitative | 65.38 |
| Kamadjeu 2017[124] | Polio | Somalia | Yes | Outcome | post-outbreak | internal | effectiveness, efficiency | primary | n/a | qualitative | 53.85 |
| Kamadjeu 2014[125] | Polio | Somalia | Yes | Outcome | post-outbreak | internal | effectiveness | mixed | output, outcome | mixed | 76.92 |
| IFRC 2010[126] | Polio | Uganda, Sudan, Côte d’Ivoire and Burkina Faso. | No | Outcome | post-outbreak | internal | n/a | mixed | outcome | mixed | 57.69 |
| Khetsuriani 2017[127] | Polio | Ukraine | Yes | Outcome | post-outbreak | internal | effectiveness | mixed | process, output, outcome | mixed | 80.77 |
| Bennet 2018[128] | Typhoid | Malawi | No | Outcome | mid-outbreak | external | effectiveness | mixed | outcome | mixed | 80.77 |
| IFRC 2013[129] | Various | Uganda | No | Outcome | post-outbreak | internal | relevance, effectiveness, accountability | mixed | process | qualitative | 65.38 |
| de la Rosa Vazquez 2017[130] | Yellow fever | DRC | Yes | Outcome | post-outbreak | internal | relevance, appropriateness and coverage; connectedness and coherence (sustainability); effectiveness; efficiency; impact; child focus; community engagement and accountability. | mixed | process, outcome | qualitative | 84.62 |
| Bagonza 2013[131] | Yellow Fever | Uganda | No | Process | post-outbreak | internal | effectiveness | mixed | process | quantitative | 84.62 |
| Ajay 2013[132] | Lassa Fever | Nigeria | No | Outcome | Post-outbreak | External | Effectiveness | Secondary | Outcome | mixed | 65.38 |

Reference

1. Malik MR, Mnzava A, Mohareb E, Zayed A, Al Kohlani A, Thabet AAK, et al. Chikungunya outbreak in Al-Hudaydah, Yemen, 2011: Epidemiological characterization and key lessons learned for early detection and control. J Epidemiol Glob Health. 2014;4:203–11.

2. Makoutodé M, Diallo F, Mongbo V, Guévart E, Bazira L. La Riposte à L’épidémie de Choléra de 2008 àCotonou (Bénin). Sante Publique (Paris). 2010;22:425–35.

3. Aka L-P, Brunnström C, Ogle M. Benin Floods, Cholera and Fire (MDRBJ 009, MDRBJ010 and MDRBJ011) DREF Review March 2013. 2013. http://adore.ifrc.org/Download.aspx?FileId=42185&.pdf.

4. Rapport de capitalisation au sujet de l’épidémie de choléra au Tchad, 2010 - Chad | ReliefWeb. https://reliefweb.int/report/chad/rapport-de-capitalisation-au-sujet-de-lépidémie-de-choléra-au-tchad-2010. Accessed 17 May 2020.

5. Grayel Y. “Programme D’Intervention Pour Limiter Et Prevenir La Propagation De L’Epidemie Du Cholera En Republique Democratique Du Congo.” ACF; 2014.

6. Ciglenecki I, Sakoba K, Luquero FJ, Heile M, Itama C, Mengel M, et al. Feasibility of Mass Vaccination Campaign with Oral Cholera Vaccines in Response to an Outbreak in Guinea. PLoS Med. 2013;10:e1001512. doi:10.1371/journal.pmed.1001512.

7. Cavallaro EC, Harris JR, Da Goia MS, Dos Santos Barrado JC, Da Nóbrega AA, De Alvarenga IC, et al. Evaluation of pot-chlorination of wells during a cholera outbreak, Bissau, Guinea-Bissau, 2008. J Water Health. 2011;9:394–402.

8. Ciglenecki I, Bichet M, Tena J, Mondesir E, Bastard M, Tran NT, et al. Cholera in Pregnancy: Outcomes from a Specialized Cholera Treatment Unit for Pregnant Women in Léogâne, Haiti. PLoS Negl Trop Dis. 2013;7.

9. Routh JA, Sreenivasan N, Adhikari BB, Andrecy LL, Bernateau M, Abimbola T, et al. Cost evaluation of a government-conducted oral cholera vaccination campaign - Haiti, 2013. American Journal of Tropical Medicine and Hygiene. 2017;97:37–42.

10. Sévère K, Rouzier V, Anglade SB, Bertil C, Joseph P, Deroncelay A, et al. Effectiveness of oral cholera vaccine in Haiti: 37-month follow-up. Am J Trop Med Hyg. 2016;94:1136–42.

11. Ivers LC, Hilaire IJ, Teng JE, Almazor CP, Jerome JG, Ternier R, et al. Effectiveness of reactive oral cholera vaccination in rural Haiti: A case-control study and bias-indicator analysis. Lancet Glob Heal. 2015;3:e162–8.

12. Santa-Olalla P, Gayer M, Magloire R, Barrais R, Valenciano M, Aramburu C, et al. Implementation of an alert and response system in Haiti during the early stage of the response to the Cholera Epidemic. Am J Trop Med Hyg. 2013;89:688–97.

13. Tauxe R V., Lynch M, Lambert Y, Sobel J, Domerçant JW, Khan A. Rapid development and use of a nationwide training program for cholera management, Haiti, 2010. Emerg Infect Dis. 2011;17:2094–8.

14. Dhillon P, Annunziata G. The Haitian Health Cluster Experience: A comparative evaluation of the professional communication response to the 2010 earthquake and the subsequent cholera outbreak. PLoS Curr. 2012;4.

15. Teng JE, Thomson DR, Lascher JS, Raymond M, Ivers LC. Using Mobile Health (mHealth) and Geospatial Mapping Technology in a Mass Campaign for Reactive Oral Cholera Vaccination in Rural Haiti. PLoS Negl Trop Dis. 2014;8:e3050. doi:10.1371/journal.pntd.0003050.

16. Grayel Y. Evaluation Externe Réponse d’Urgence à L’Epidémie de Choléra en Haïti (ACF). 2011.

17. Dobai A, Tallada J. Final Evaluation of the Cholera Emergency Appeal in Haiti and the Dominican Republic. 2016. https://www.ifrc.org/en/publications-and-reports/evaluations/?c=&co=&fy=&mo=&mr=1&or=&r=&ti=haiti&ty=&tyr=&z=. Accessed 24 Jul 2019.

18. Tappero JW, Tauxe R V. Lessons learned during public health response to cholera epidemic in Haiti and the Dominican Republic. Emerg Infect Dis. 2011;17:2087–93.

19. Khonje A, Metcalf CA, Diggle E, Mlozowa D, Jere C, Akesson A, et al. Cholera outbreak in districts around Lake Chilwa, Malawi: Lessons learned. Malawi Med J. 2012;24:29–33.

20. Msyamboza KP, M’bang’ombe M, Hausi H, Chijuwa A, Nkukumila V, Kubwalo HW, et al. Feasibility and acceptability of oral cholera vaccine mass vaccination campaign in response to an outbreak and floods in Malawi. Pan Afr Med J. 2016;23:203.

21. Oladele DA, Oyedeji KS, Niemogha MT, Nwaokorie F, Bamidele M, Musa AZ, et al. An assessment of the emergency response among health workers involved in the 2010 cholera outbreak in northern Nigeria. J Infect Public Health. 2012;5:346–53. doi:http://dx.doi.org/10.1016/j.jiph.2012.06.004.

22. Fogden D, Matoka S, Singh G. MDRNG020 Nigeria Cholera Epidemic Operational Review. Nigeria; 2016. https://www.ifrc.org/en/publications-and-reports/evaluations/?c=&co=&fy=&mo=&mr=1&or=&r=&ti=nigeria&ty=&tyr=&z=. Accessed 24 Jun 2019.

23. Peter Rees-Gildea. Sierra Leone Cholera ERU Operation Review | ALNAP. 2013. https://www.alnap.org/help-library/sierra-leone-cholera-eru-operation-review. Accessed 17 May 2020.

24. Evaluation of Sierra Leone Cholera Response 2012 Project Effectiveness Review Oxfam GB Global Humanitarian Indicator. 2013.

25. Mcgowan C. Somalia OCV campaign After Action Review. 2018.

26. Dyson C. EHU After Action Review Somalia Cholera Response 2017. 2018.

27. Azman AS, Parker LA, Rumunu J, Tadesse F, Grandesso F, Deng LL, et al. Effectiveness of one dose of oral cholera vaccine in response to an outbreak: a case-cohort study. Lancet Glob Heal. 2016;4:e856–63.

28. Gauthier J. A Real-Time Evaluation of ACF’s response to cholera emergency in Juba, South Sudan | ALNAP. 2014. https://www.alnap.org/help-library/a-real-time-evaluation-of-acf’s-response-to-cholera-emergency-in-juba-south-sudan. Accessed 17 May 2020.

29. Bwire G, Mwesawina M, Baluku Y, Kanyanda SSE, Orach CG. Cross-border cholera outbreaks in Sub-Saharan Africa, the mystery behind the silent illness: What needs to be done? PLoS One. 2016;11.

30. Dureab F, Ismail O, Müller O, Jahn A. Cholera outbreak in Yemen: Timeliness of reporting and response in the national electronic disease early warning system. Acta Inform Medica. 2019;27:85–8.

31. Altmann M, Suarez-Bustamante M, Soulier C, Lesavre C, Antoine C. First Wave of the 2016-17 Cholera Outbreak in Hodeidah City, Yemen - Acf Experience and Lessons Learned. PLoS Curr. 2017.

32. Federspiel F, Ali M. The cholera outbreak in Yemen: Lessons learned and way forward. BMC Public Health. 2018;18:1338. doi:10.1186/s12889-018-6227-6.

33. Darcy J, Valingot C, Olsen L, Noor al deen A, Qatinah A. A crisis within a crisis -evaluation of the UNICEF Level 3 response to the cholera epidemic in Yemen. 2018. https://www.unicef.org/evaldatabase/files/Evaluation_of_the_UNICEF_Level_3_response_to_the_cholera_epidemic_in_Yemen_HQEO-2018-001.pdf. Accessed 18 Apr 2019.

34. Spiegel P, Ratnayake R, Hellman N, Lantagne D, Ververs M, Ngwa M, et al. I Cholera in Yemen: a case study of epidemic preparedness and response CHOLERA IN YEMEN: A CASE STUDY OF EPIDEMIC PREPAREDNESS AND RESPONSE. 2019.

35. Ashbaugh HR, Kuang B, Gadoth A, Alfonso VH, Mukadi P, Doshi RH, et al. Detecting Ebola with limited laboratory access in the Democratic Republic of Congo: evaluation of a clinical passive surveillance reporting system. Trop Med Int Heal. 2017;22:1141–53.

36. McGowan C. AFTER ACTION REVIEW DRC EVD Response in North Kivu and Ituri 2019. 2019.

37. Hennessee I, Guilavogui T, Camara A, Halsey ES, Marston B, McFarland D, et al. Adherence to Ebola-specific malaria case management guidelines at health facilities in Guinea during the West African Ebola epidemic. Malar J. 2018;17.

38. Diallo A, Diallo M, Hyjazi Y, Waxman R, Pleah T. Baseline evaluation of infection prevention and control (IPC) in the context of Ebola virus disease (EVD) in nine healthcare facilities in the city of Conakry, Guinea. Antimicrob Resist Infect Control. 2015;4:1–1.

39. Fu C, Roberton T, Burnham G. Community-based social mobilization and communications strategies utilized in the 2014 West Africa Ebola outbreak. Ann Glob Heal. 2015;81:126.

40. Standley CJ, Muhayangabo R, Bah MS, Barry AM, Bile E, Fischer JE, et al. Creating a National Specimen Referral System in Guinea: Lessons From Initial Development and Implementation. Front Public Heal. 2019;7 MAR:83. doi:10.3389/fpubh.2019.00083.

41. Lee CT, Bulterys M, Martel, LD, Dahl BA. Evaluation of a National Call Center and a Local Alerts System for Detection of New Cases of Ebola Virus Disease — Guinea, 2014–2015. MMWR Morb Mortal Wkly Rep. 2016;65:227–30. doi:10.15585/mmwr.mm6509a2.

42. Requesa L, Bolibarb I, Chazelleb E, Gomesb L, Prikazsky V, Banza F, et al. Evaluation of contact tracing activities during the Ebola virus disease outbreak in Guinea, 2015. Int Health. 2017;9:131–3.

43. Soeters HM, Koivogui L, de Beer L, Johnson CY, Diaby D, Ouedraogo A, et al. Infection prevention and control training and capacity building during the Ebola epidemic in Guinea. PLoS One. 2018;13.

44. Kokki M, Safrany N. Evaluation of ECDC Ebola deployment in Guinea Final report Evaluation of ECDC Ebola deployment in Guinea. Stockholm; 2017. doi:10.2900/202126.

45. Fitzpatrick G, Decroo T, Draguez B, Crestani R, Ronsse A, Van den Bergh R, et al. Operational research during the Ebola emergency. Emerg Infect Dis. 2017;23:1057–62.

46. Murray A, Majwa P, Roberton T, Burnham G. Report of the real time evaluation of Ebola control programs in Guinea, Sierra Leone and Liberia | ALNAP. 2015. https://www.alnap.org/help-library/report-of-the-real-time-evaluation-of-ebola-control-programs-in-guinea-sierra-leone-and. Accessed 17 May 2020.

47. Global: Evaluation of UNICEF’s response to the Ebola outbreak in West Africa, 2014-2015 | Evaluation database | UNICEF. 2016. https://www.unicef.org/evaldatabase/index_95016.html. Accessed 17 May 2020.

48. WHO | Report of the Ebola Interim Assessment Panel - July 2015. WHO. 2020.

49. Shepherd M, Frize J, De Meulder F, Bizzari M, Lemaire I, Horst LR, et al. An evaluation of WFP’s L3 Response to the Ebola virus disease (EVD) crisis in West Africa Evaluation Report WFP Office of Evaluation. 2017.

50. with Jennifer Leigh S, Cook G, Hansch S, Toole Swati Sadaphal M, -Team Leader Jennifer Leigh M, -Public Health Advisor Gayla Cook M, et al. Evaluation of the USAID/OFDA Ebola Virus Disease Outbreak Response in West Prepared for. 2017.

51. Swati Sadaphal with, Leigh J, Toole M, Cook Swati Sadaphal G, -Team Leader Jennifer Leigh M, -Public Health Advisor Gayla Cook M, et al. Evaluation of the USAID/OFDA Ebola Virus Disease Outbreak Response in West Africa 2014-2016 Objective 4: Coordination of the Response USAID/DCHA/OFDA CONTRACT # AID-OAA-I-15-00022 Task Order # AID-OAA-TO-16-00034 Prepared for.

52. Cook G, Leigh J, Toole M, Hansch S, Sadaphal S, Leader M-T, et al. Evaluation of the USAID/OFDA Ebola Virus Disease Outbreak Response in West Africa 2014-2016 Objective 2: Effectiveness of Programmatic Components. Effectiveness of Programmatic; 2018.

53. of Commons H. Ebola: Responses to a public health emergency Second Report of Session 2015-16 HC 338.

54. Lupel A, Snyder M. The Mission to Stop Ebola: Lessons for UN Crisis Response. 2017. www.ipinst.org. Accessed 18 May 2020.

55. Bell BP, Damon IK, Jernigan DB, Kenyon TA, Nichol ST, O’Connor JP, et al. Overview, Control Strategies, and Lessons Learned in the CDC Response to the 2014–2016 Ebola Epidemic. MMWR Suppl. 2016;65:4–11. doi:10.15585/mmwr.su6503a2.

56. Summers A, Nyensaw T, Montgomery JM, Neatherlin J, Tappero JW. Challenges in Responding to the Ebola Epidemic — Four Rural Counties, Liberia, August–November 2014. MMWR Recomm reports Morb Mortal Wkly report Recomm reports. 2014;63:1202–4. https://www.cdc.gov/mmwr/preview/mmwrhtml/mm6350a5.htm. Accessed 16 May 2020.

57. Swanson KC, Altare C, Wesseh CS, Nyenswah T, Ahmed T, Eyal N, et al. Contact tracing performance during the Ebola epidemic in Liberia, 2014-2015. PLoS Negl Trop Dis. 2018;12:e0006762. doi:10.1371/journal.pntd.0006762.

58. Kateh F, Nagbe T, Kieta A, Barskey A, Gasasira AN, Driscoll A, et al. Rapid response to ebola outbreaks in remote areas — Liberia, July–November 2014. Morb Mortal Wkly Rep. 2015;64:188–92.

59. Cardile AP, Littell CT, Backlund MG, Heipertz RA, Brammer JA, Palmer SM, et al. Deployment of the 1st Area Medical Laboratory in a Split-Based Configuration During the Largest Ebola Outbreak in History. Mil Med. 2016;181:e1675–84.

60. Emmie de Wit, Kyle Rosenke, Robert J. Fischer, Andrea Marzi, Joseph Prescott, Trenton Bushmaker, et al. Ebola Laboratory Response at the Eternal Love Winning Africa Campus, Monrovia, Liberia, 2014–2015. J Infect Dis. 2016;214 Suppl 3:S169–76. https://www.ncbi.nlm.nih.gov/pmc/articles/PMC5050467/. Accessed 16 May 2020.

61. Wolfe CM, Hamblion EL, Schulte J, Williams P, Koryon A, Enders J, et al. Ebola virus disease contact tracing activities, lessons learned and best practices during the Duport Road outbreak in Monrovia, Liberia, November 2015. PLoS Negl Trop Dis. 2017;11.

62. Katawera V, Kohar H, Mahmoud N, Raftery P, Wasunna C, Humrighouse B, et al. Enhancing laboratory capacity during Ebola virus disease (EVD) heightened surveillance in Liberia: lessons learned and recommendations. Pan Afr Med J. 2019;33:8.

63. Kouadio KI, Clement P, Bolongei J, Tamba A, Gasasira AN, Warsame A, et al. Epidemiological and surveillance response to ebola virus disease outbreak in lofa county, liberia (march-september, 2014); lessons learned. PLoS Curr. 2015;7 OUTBREAKS.

64. Sepers CE, Fawcett SB, Hassaballa I, Reed FD, Schultz J, Munodawafa D, et al. Evaluating implementation of the Ebola response in Margibi County, Liberia. Health Promot Int. 2019;34:510–2.

65. Oji MO, Haile M, Baller A, Tremblay N, Mahmoud N, Gasasira A, et al. Implementing infection prevention and control capacity building strategies within the context of Ebola outbreak in a “Hard-to-Reach” area of Liberia. Pan Afr Med J. 2018;31.

66. Nyenswah T, Engineer CY, Peters DH. Leadership in Times of Crisis: The Example of Ebola Virus Disease in Liberia. Heal Syst Reform. 2016;2:194–207. doi:10.1080/23288604.2016.1222793.

67. Munodawafa D, Moeti MR, Phori PM, Fawcett SB, Hassaballa I, Sepers C, et al. Monitoring and Evaluating the Ebola Response Effort in Two Liberian Communities. J Community Health. 2018;43:321–7.

68. Nevin RL, Anderson JN. The timeliness of the US military response to the 2014 Ebola disaster: a critical review. Med Confl Surviv. 2016;32:40–69.

69. Abramowitz S, Bardosh K, Heaner G. Evaluation of Save the Children’s Community Care Centers in Dolo Town and Worhn, Margibi County, Liberia | ALNAP. 2015. https://www.alnap.org/help-library/evaluation-of-save-the-childrens-community-care-centers-in-dolo-town-and-worhn-margibi. Accessed 17 May 2020.

70. Evaluation Report | Liberia YMCA Ebola Outbreak Emergency Response Learning for impact Humanitarian Response Executive Summary Acknowledgements from Liberia YMCA. 2015.

71. Global Communities. Stopping Ebola in its Tracks: A Community-Led Response. 2015.

72. Carafano JJ, Florance C, Kaniewski D. The Ebola Outbreak of 2013–2014: An Assessment of U.S. Actions | The Heritage Foundation. 2015. https://www.heritage.org/homeland-security/report/the-ebola-outbreak-2013-2014-assessment-us-actions. Accessed 18 May 2020.

73. Clive M. Brown, Aaron E. Aranas, Gabrielle A. Benenson, , Gary Brunette, Marty Cetron, Tai-Ho Chen, Nicole J. Cohen, Pam Diaz, Yonat Haber, Christa R. Hale, Kelly Holton, Katrin Kohl, MD1, Amanda W. Lee, MPH1, Gabriel J. Palumbo, Kate Pearson, Christina R NP. Airport Exit and Entry Screening for Ebola — August–November 10, 2014. https://www.cdc.gov/mmwr/preview/mmwrhtml/mm6349a5.htm. Accessed 16 Feb 2020.

74. Hurtado C, Meyer D, Snyder M, Nuzzo JB. Evaluating the frequency of operational research conducted during the 2014–2016 West Africa Ebola epidemic. Int J Infect Dis. 2018;77:29–33.

75. Elemuwa C, Kutalek R, Ali M, Mworozi E, Kochhar S, Rath B, et al. Global lessons from Nigeria’s ebolavirus control strategy. Expert Rev Vaccines. 2015;14:1397–400. doi:10.1586/14760584.2015.1064313.

76. Oleribe OO, Crossey MME, Taylor-Robinson SD. Nigerian response to the 2014 Ebola viral disease outbreak: lessons and cautions. Pan Afr Med J. 2015;22 Suppl 1:13.

77. Asuzu MC, Onajole AT, Disu Y. Public health at all levels in the recent Nigerian Ebola viral infection epidemic: Lessons for community, public and international health action and policy. J Public Health Policy. 2015;36:251–8.

78. Vaz RG, Mkanda P, Banda R, Komkech W, Ekundare-Famiyesin OO, Onyibe R, et al. The Role of the Polio Program Infrastructure in Response to Ebola Virus Disease Outbreak in Nigeria 2014. J Infect Dis. 2016;213:S140–6. https://www.ncbi.nlm.nih.gov/pmc/articles/PMC4818557/. Accessed 17 May 2020.

79. Li ZJ, Tu WX, Wang XC, Shi GQ, Yin ZD, Su HJ, et al. A practical community-based response strategy to interrupt Ebola transmission in sierra Leone, 2014-2015. Infect Dis Poverty. 2016;5.

80. Ratnayake R, Crowe SJ, Jasperse J, Privette G, Stone E, Miller L, et al. Assessment of community event-based surveillance for Ebola virus disease, Sierra Leone, 2015. Emerg Infect Dis. 2016;22:1431–7.

81. Youkee D, Brown CS, Lilburn P, Shetty N, Brooks T, Simpson A, et al. Assessment of Environmental Contamination and Environmental Decontamination Practices within an Ebola Holding Unit, Freetown, Sierra Leone. PLoS One. 2015;10.

82. Vogt F, Fitzpatrick G, Patten G, van den Bergh R, Stinson K, Pandolfi L, et al. Assessment of the MSF triage system, separating patients into different wards pending ebola virus laboratory confirmation, Kailahun, Sierra Leone, July to September 2014. Eurosurveillance. 2015;20.

83. Logue CH, Lewis SM, Lansley A, Fraser S, Shieber C, Shah S, et al. Case study: Design and implementation of training for scientists deploying to ebola diagnostic field laboratories in Sierra Leone: October 2014 to February 2016. Philos Trans R Soc B Biol Sci. 2017;372.

84. Stone E, Miller L, Jasperse J, Privette G, Diez Beltran JC, Jambai A, et al. Community Event-Based Surveillance for Ebola Virus Disease in Sierra Leone: Implementation of a National-Level System During a Crisis. PLoS Curr. 2016;8.

85. Olu OO, Lamunu M, Nanyunja M, Dafae F, Samba T, Sempiira N, et al. Contact Tracing during an Outbreak of Ebola Virus Disease in the Western Area Districts of Sierra Leone: Lessons for Future Ebola Outbreak Response. Front Public Heal. 2016;4.

86. Senga M, Koi A, Moses L, Wauquier N, Barboza P, Fernandez-Garcia MD, et al. Contact tracing performance during the ebola virus disease outbreak in kenema district, Sierra Leone. Philos Trans R Soc B Biol Sci. 2017;372.

87. Oza S, Jazayeri D, Teich JM, Ball E, Nankubuge PA, Rwebembera J, et al. Development and deployment of the OpenMRS-Ebola electronic health record system for an Ebola treatment center in Sierra Leone. J Med Internet Res. 2017;19.

88. Jobanputra K, Greig J, Shankar G, Perakslis E, Kremer R, Achar J, et al. Electronic medical records in humanitarian emergencies - the development of an Ebola clinical information and patient management system. F1000Research. 2017;5.

89. Gleason B, Redd J, Kilmarx P, Sesay T, Bayor F, Mozalevskis A, et al. Establishment of an ebola treatment unit and laboratory — Bombali District, Sierra Leone, July 2014–January 2015. Morb Mortal Wkly Rep. 2015;64:1108–11.

90. Jia K, Mohamed K. Evaluating the use of cell phone messaging for community ebola syndromic surveillance in high risked settings in Southern Sierra Leone. Afr Health Sci. 2015;15:797–802.

91. Ilesanmi OS, Fawole O, Nguku P, Oladimeji A, Nwenyi O. Evaluation of Ebola virus disease surveillance system in Tonkolili District, Sierra Leone. Pan Afr Med J. 2019;32 Suppl 1:2.

92. Garde DL, Hall AMR, Marsh RH, Barron KP, Dierberg KL, Koroma AP. Implementation of the first dedicated Ebola screening and isolation for maternity patients in Sierra Leone. Ann Glob Heal. 2016;82:418. doi:10.1016/j.aogh.2016.04.164.

93. Nielsen CF, Kidd S, Sillah ARM, Davis E, Mermin J, Kilmarx PH. Improving burial practices and cemetery management during an Ebola virus disease epidemic — Sierra Leone, 2014. Morb Mortal Wkly Rep. 2015;64:20–7.

94. Nic Lochlainn LM, Gayton I, Theocharopoulos G, Edwards R, Danis K, Kremer R, et al. Improving mapping for Ebola response through mobilising a local community with self-owned smartphones: Tonkolili District, Sierra Leone, January 2015. PLoS One. 2018;13:e0189959. doi:10.1371/journal.pone.0189959.

95. Jones-Konneh TEC, Murakami A, Sasaki H, Egawa S. Intensive education of health care workers improves the outcome of ebola virus disease: Lessons learned from the 2014 outbreak in Sierra Leone. Tohoku Journal of Experimental Medicine. 2017;243:101–5.

96. Hermans V, Zachariah R, Woldeyohannes D, Saffa G, Kamara D, Ortuno-Gutierrez N, et al. Offering general pediatric care during the hard times of the 2014 Ebola outbreak: Looking back at how many came and how well they fared at a Médecins Sans Frontières referral hospital in rural Sierra Leone. BMC Pediatr. 2017;17.

97. Cancedda C, Davis SM, DIerberg KL, Lascher J, Kelly JD, Barrie MB, et al. Strengthening Health Systems while Responding to a Health Crisis: Lessons Learned by a Nongovernmental Organization during the Ebola Virus Disease Epidemic in Sierra Leone. J Infect Dis. 2016;214:S153–63.

98. Lokuge K, Caleo G, Greig J, Duncombe J, McWilliam N, Squire J, et al. Successful Control of Ebola Virus Disease: Analysis of Service Based Data from Rural Sierra Leone. PLoS Negl Trop Dis. 2016;10.

99. Stehling-Ariza T, Rosewell A, Moiba SA, Yorpie BB, Ndomaina KD, Jimissa KS, et al. The impact of active surveillance and health education on an Ebola virus disease cluster - Kono District, Sierra Leone, 2014-2015. BMC Infect Dis. 2016;16:611. doi:10.1186/s12879-016-1941-0.

100. Miller LA, Stanger E, Senesi RG, DeLuca N, Dietz P, Hausman L, et al. Use of a Nationwide Call Center for Ebola Response and Monitoring During a 3-Day House-to-House Campaign — Sierra Leone, September 2014. MMWR Morb Mortal Wkly Rep. 2015;64:28–9. https://www.cdc.gov/mmwr/preview/mmwrhtml/mm6401a7.htm. Accessed 17 May 2020.

101. Fearon C. Humanitarian Quality Assurance - Sierra Leone: Evaluation of Oxfam’s humanitarian response to the West Africa Ebola crisis | Oxfam Policy & Practice. 2017. https://policy-practice.oxfam.org.uk/publications/humanitarian-quality-assurance-sierra-leone-evaluation-of-oxfams-humanitarian-r-620191. Accessed 17 May 2020.

102. Sierra Leone YMCA Ebola Outbreak Emergency Response - Evaluation report, February 2016 - Sierra Leone | ReliefWeb. 2016. https://reliefweb.int/report/sierra-leone/sierra-leone-ymca-ebola-outbreak-emergency-response-evaluation-report-february. Accessed 17 May 2020.

103. Cascioli Sharp R. Real-time learning report on World Vision’s response to the ebola virus in Sierra Leone | ALNAP. 2015. https://www.alnap.org/help-library/real-time-learning-report-on-world-visions-response-to-the-ebola-virus-in-sierra-leone. Accessed 17 May 2020.

104. Platt A, Kerley L. External Evaluation of Plan International UK’s Response to the Ebola Virus Outbreak in Sierra Leone | ALNAP. 2016. https://www.alnap.org/help-library/external-evaluation-of-plan-international-uk’s-response-to-the-ebola-virus-outbreak-in. Accessed 18 May 2020.

105. Bayntun C, Zimble SA. Evaluation of the OCG Response to the Ebola Outbreak Lessons learned from the Freetown Ebola Treatment Unit, Sierra Leone Managed by the Vienna Evaluation Unit. 2016. http://tukul.msf.org. Accessed 18 May 2020.

106. TKG International. CARE International DEC Ebola Emergency Response Project Final Evaluation Report. 2016. www.thekhanagroup.com. Accessed 18 May 2020.

107. Momoh HB, Lamin F, Samai I. Final Report: Evaluation of DEC Ebola Response Program Phase 1 and 2 DEC Emergency Response Program Implemented by CAFOD, Caritas, Street Child and Troacaire in Sierra Leone. 2016.

108. Evaluation of Disasters Emergency Committee and Age International funded: Responding to the Ebola outbreak in Sierra Leone through age-inclusive community-led action . 2015. https://www.alnap.org/help-library/evaluation-of-disasters-emergency-committee-and-age-international-funded-responding-to. Accessed 18 May 2020.

109. Adams J, Lloyd A, Miller C. The Oxfam Ebola Response in Liberia and Sierra Leone: An evaluation report for the Disasters Emergency Committee | Oxfam Policy & Practice. 2015. https://policy-practice.oxfam.org.uk/publications/the-oxfam-ebola-response-in-liberia-and-sierra-leone-an-evaluation-report-for-t-560602. Accessed 17 May 2020.

110. Borchert M, Mutyaba I, Van Kerkhove MD, Lutwama J, Luwaga H, Bisoborwa G, et al. Ebola haemorrhagic fever outbreak in Masindi District, Uganda: Outbreak description and lessons learned. BMC Infectious Diseases. 2011;11.

111. Mbonye AK, Wamala JF, Nanyunja M, Opio A, Aceng JR, Makumbi I. Ebola viral hemorrhagic disease outbreak in West Africa- lessons from Uganda. Afr Health Sci. 2014;14:495–501.

112. Thormar B S. Joint review of Ebola response – Uganda. Uganda; 2013. https://www.ifrc.org/en/publications-and-reports/evaluations/?c=&co=&fy=&mo=&mr=1&or=&r=&ti=uganda&ty=&tyr=&z=. Accessed 24 May 2019.

113. Abubakar A, Ruiz-Postigo JA, Pita J, Lado M, Ben-Ismail R, Argaw D, et al. Visceral Leishmaniasis Outbreak in South Sudan 2009-2012: Epidemiological Assessment and Impact of a Multisectoral Response. PLoS Negl Trop Dis. 2014;8.

114. A.S. W, B.G. M, G. G, J.L. G, H. B, M. A, et al. Evaluation of economic costs of a measles outbreak and outbreak response activities in Keffa Zone, Ethiopia. Vaccine. 2014;32:4505–14. doi:10.1016/j.vaccine.2014.06.035 LK - http://findit.library.jhu.edu/resolve?sid=EMBASE&issn=18732518&id=doi:10.1016%2Fj.vaccine.2014.06.035&atitle=Evaluation+of+economic+costs+of+a+measles+outbreak+and+outbreak+response+activities+in+Keffa+Zone%2C+Ethiopia&stitle=Vaccine&title=Vaccine&volume=32&issue=35&spage=4505&epage=4514&aulast=Wallace&aufirst=Aaron+S.&auinit=A.S.&aufull=Wallace+A.S.&coden=VACCD&isbn=&pages=4505-4514&date=2014&auinit1=A&auinitm=S.

115. Lowe T. Emergency Health Unit After Action Review Madagascar July 2019. 2019.

116. Ntshoe GM, McAnerney JM, Archer BN, Smit SB, Harris BN, Tempia S, et al. Measles Outbreak in South Africa: Epidemiology of Laboratory-Confirmed Measles Cases and Assessment of Intervention, 2009–2011. PLoS One. 2013;8.

117. Yaméogo TM, Kyelem CG, Poda GEA, Sombié I, Ouédraogo MS, Millogo A. Épidémie de méningite : Évaluation de la surveillance et du traitement des cas dans les formations sanitaires d’un district du Burkina Faso. Bull la Soc Pathol Exot. 2011;104:68–73.

118. Heitzinger K, Impouma B, Farham B, Hamblion EL, Lukoya C, MacHingaidze C, et al. Using evidence to inform response to the 2017 plague outbreak in Madagascar: A view from the WHO African Regional Office. Epidemiol Infect. 2019;147:e3. doi:http://dx.doi.org/10.1017/S0950268818001875.

119. Ndiaye SM, Ahmed MA, Denson M, Craig AS, Kretsinger K, Cherif B, et al. Polio Outbreak Among Nomads in Chad: Outbreak Response and Lessons Learned. J Infect Dis. 2014;210.

120. Tegegne AA, Braka F, Shebeshi ME, Aregay AK, Beyene B, Mersha AM, et al. Characteristics of wild polio virus outbreak investigation and response in Ethiopia in 2013-2014: implications for prevention of outbreaks due to importations. BMC Infect Dis. 2018;18:9.

121. Victoria M. Gammino, Adamu Nuhu, Sue Gerber, Alex Gasasira, David E. Sugerman, Fadinding Manneh, Paul Chenoweth, Molly R. Kurnit EAA. Evaluation of Polio Supplemental Immunization Activities in Kano, Katsina, and Zamfara States, Nigeria: Lessons in Progress | The Journal of Infectious Diseases | Oxford Academic. J Infect Dis. 2014;20:S91–7. https://academic.oup.com/jid/article/210/suppl_1/S91/2194397. Accessed 16 May 2020.

122. Nkwogu L, Shuaib F, Braka F, Mkanda P, Banda R, Korir C, et al. Impact of engaging security personnel on access and polio immunization outcomes in security-inaccessible areas in Borno state, Nigeria. BMC Public Health. 2018;18:1311. doi:10.1186/s12889-018-6188-9.

123. Adamu US, Archer WR, Braka F, Damisa E, Siddique A, Baig S, et al. Progress toward poliomyelitis eradication — Nigeria, January 2018–May 2019. Morbidity and Mortality Weekly Report. 2019;68:642–6.

124. Kamadjeu R, Gathenji C. Designing and implementing an electronic dashboard for disease outbreaks response - Case study of the 2013-2014 Somalia Polio outbreak response dashboard. Pan Afr Med J. 2017;27:22.

125. Kamadjeu R, Mahamud A, Webeck J, Baranyikwa MT, Chatterjee A, Bile YN, et al. Polio outbreak investigation and response in Somalia, 2013. J Infect Dis. 2014;210 Suppl:S181-6. doi:https://dx.doi.org/10.1093/infdis/jiu453.

126. IFRC. Evaluation of the Red Cross and Red Crescent contribution to the 2009 Africa polio outbreak response. 2010.

127. Khetsuriani N, Perehinets I, Nitzan D, Popovic D, Moran T, Allahverdiyeva V, et al. Responding to a cVDPV1 outbreak in Ukraine: Implications, challenges and opportunities. Vaccine. 2017;35:4769–76.

128. Bennett SD, Lowther SA, Chingoli F, Chilima B, Kabuluzi S, Ayers TL, et al. Assessment of water, sanitation and hygiene interventions in response to an outbreak of typhoid fever in Neno District, Malawi. PLoS One. 2018;13.

129. IFRC. Health Epidemics Joint Evaluation Report (IFRC). Uganda; 2013. https://www.ifrc.org/en/publications-and-reports/evaluations/?c=&co=&fy=&mo=&mr=1&or=&r=&ti=joint evaluation&ty=&tyr=&z=. Accessed 24 Jul 2019.

130. de la Rosa Vazquez O. Evaluation of EHU/Save the Children Democratic Republic of Congo (DRC) Yellow Fever Mass Vaccination Campaign in Binza Ozone Health Zone, Kinshasa province (DRC), 2016. 2017.

131. Bagonza J, Rutebemberwa E, Mugaga M, Tumuhamye N, Makumbi I. Yellow fever vaccination coverage following massive emergency immunization campaigns in rural Uganda, May 2011: A community cluster survey. BMC Public Health. 2013;13.

132. Ajayi NA, Nwigwe CG, Azuogu BN, Onyire BN, Nwonwu EU, Ogbonnaya LU, et al. Containing a Lassa fever epidemic in a resource-limited setting: Outbreak description and lessons learned from Abakaliki, Nigeria (January-March 2012). Int J Infect Dis. 2013;17:e1011–6. doi:10.1016/j.ijid.2013.05.015.
