# Supplementary material for: The practice of evaluating epidemic response in humanitarian and low-income settings: a systematic review
Source: BMC Med. 2020 Nov 3;18:315. doi: 10.1186/s12916-020-01767-8 (PMC7606030; doi:10.1186/s12916-020-01767-8)
Supplement: Supplementary file 3 — Additional file 3. Search Strategy & Evaluation Quality Checklist. [file 12916_2020_1767_MOESM3_ESM.docx]

### Evaluation Quality Checklist

| **Criterion** | | **Sub-score** | **Weight** (W) |
| --- | --- | --- | --- |
|  |  | (S) |  |
| **1** | **Scope** | | 0..25 |
|  | Rationale of evaluation clearly stated |  |  |
|  | specifies the evaluation criteria against which the subject to be evaluated be assessed, including, for example, relevance, efficiency, effectiveness, impact and/or sustainability |  |  |
|  | includes a comprehensive and tailored evaluation framework including evaluation criteria, questions and required indicators |  |  |
|  | Gives clear and relevant description of the context of the intervention (policy, socioeconomic, political, institutional, international factors relevant to the implementation of the intervention). |  |  |
|  | The geographic context and boundaries are clearly mentioned |  |  |
| **2** | **Methodology** | | 0.25 |
|  | Contains a clear and complete description of a relevant design and methodological approach that are suitable for the evaluation's purpose, objectives and scope |  |  |
|  | Existing information sources, such as monitoring systems and/or previous evaluations are identified. An appraisal of quality and reliability is provided. |  |  |
|  | The evaluation methodology includes multiple methods (triangulation); preferably with analysis of both quantitative and qualitative data and with a range of stakeholders covered by the data collection methods |  |  |
|  | The evaluation methodology explicitly and clearly states the limitations of the chosen evaluation methods. |  |  |
|  |  |  |  |
|  | describes the key stages of the evaluation process and the implementation time line |  |  |
| **3** | **Findings** | | 0.25 |
|  | Reported findings address the evaluation criteria (such as efficiency, effectiveness, sustainability, impact and relevance) and questions defined in the evaluation scope. |  |  |
|  | Findings are objectively reported based on the evidence. |  |  |
| **4** | **Recommendations** | | 0.25 |
|  | Recommendations are relevant to the object and purposes of the evaluation, are supported by evidence and conclusions, and were developed with the involvement of relevant stakeholders. |  |  |
| **Summary Quality Score** = (S_1_ x W_1_) + (S_2_ x W_2_) + (S_3_ x W_3_) + (S_4_ x W_4_)) | | | |

**Scoring :** **1**- Criteria fully demonstrated, **0.5** -Criteria partially demonstrated, **0** – no evidence of criteria demonstrated

## Annex

### Epidemic Search

The following sources were reviewed in order to compile a list of reported epidemics: World Health Organization Disease Outbreak News (WHO DON), WHO weekly epidemiological monitor (WEM), WHO EMRO Weekly Epidemiological Record (WER), UNICEF Cholera platform, Reliefweb, PROMED and Global Incidence Map.

In line with WHO guidance on communicable disease control in emergencies one suspected case of the following was considered to be an epidemic requiring a response: acute haemorrhagic fevers (Ebola, Lassa fever, Rift valley fever, Crimea-Congo haemorrhagic fever), anthrax, cholera, measles, typhus, plague and polio. For the remainder of the pathogens, we defined an epidemic as an unusual increase in incidence.

WHO DON reports were stored as narratives requiring each report to be opened and the metadata on location (country), year, month, pathogen extracted.

Reliefweb was searched for reported epidemics using the search engine and the disaster type filter set to find epidemics. Reports of epidemics were assessed for the period 2010-2019. Metadata on location (country), year, month, pathogen were extracted.

For the PROMED database, only epidemics rated as 3 or higher in the 5-point rating system and in which incident cases and deaths were reported were considered for inclusion. Metadata on location (country), year, month, pathogen were extracted for the period 2010-2019.

Global Incident Map database was searched utilizing the inbuilt search function filtering results that were out of scope (wrong location, pathogen etc.) at the source. Metadata on location (country), year, month, pathogen were extracted.

We collated all epidemic records into a single database. We sorted by date (month and year) and location(country). We removed duplicate reports of an epidemic from a single source (e.g. an update on an earlier reported epidemic) as well as removed duplicate reports of a single epidemic reported amongst multiple sources. In the absence of data which would have precisely determined the linkage between epidemics within a geographic boundary (e.g. genomic sequencing of epidemic pathogens) we developed a decision rule to further refine and summarize the remaining reported epidemics. We utilized a decision rule in which reports of epidemics caused by a single pathogen occurring within a 4-month window and within the boundaries of a single country were viewed as a single epidemic.

### Search terms

Medline search

1. Outbreak Terms

(Cholera OR Dysentery OR Diarrh*OR conjunctivitis OR dengue OR diphtheria OR viral hepatitis, Japanese encephalitis OR leishmaniosis OR malaria OR measles OR Meningococcal meningitis OR African sleeping sickness OR Typhoid OR Typhus OR yellow fever OR viral haemorrhagic fevers OR Communicable disease* OR Infectious disease*) adj2 (outbreak* OR epidemic* OR pandemic*)

1. Humanitarian terms

Humanitarian adj2 (cris#s OR emergenc* OR disaster* OR relief OR aid) OR humanitarian OR cris#s OR emergenc* OR disaster* OR relief OR aid OR refugee* OR evacuee OR evacuated OR displace* adj2 (population or internal*) OR war OR war adj2 (armed OR zone) OR conflict adj2 (armed OR zone) OR conflict affected adj3 (population OR communit*) OR earthquake OR flood* OR landslide* OR tidal waves OR tsunami* OR cyclon* OR typhoon* OR drought OR fragile state

1. Evaluation terms

(Evaluation* OR assessment* OR appraisal* OR Guidance* OR Lesson*)

| Citation (author, year) | Type of pathogen | Country | Humanitarian setting? (Y/N) | Type of document (guidance, evaluation report etc) | Type of evaluation (process, impact etc) | Timing (mid outbreak or post outbreak) | Commissioned by ( e.g. internal/external or donor driven) | External or internal implementation | Evaluation framework if used | Type of data collection (primary or secondary) recommended or utilized | Type of indicators of focus | Type of data collection (Quant vs qual) utilized | Quality Score |
| --- | --- | --- | --- | --- | --- | --- | --- | --- | --- | --- | --- | --- | --- |
|  |  |  |  |  |  |  |  |  |  |  |  |  |  |
|  |  |  |  |  |  |  |  |  |  |  |  |  |  |
|  |  |  |  |  |  |  |  |  |  |  |  |  |  |
|  |  |  |  |  |  |  |  |  |  |  |  |  |  |
|  |  |  |  |  |  |  |  |  |  |  |  |  |  |
|  |  |  |  |  |  |  |  |  |  |  |  |  |  |
|  |  |  |  |  |  |  |  |  |  |  |  |  |  |

### Extraction Table

### Search Strategy- Medline, Embase and Global Health

1. (outbreak* or epidemic* or pandemic*).mp. [mp=title, abstract, original title, name of substance word, subject heading word, floating sub-heading word, keyword heading word, organism supplementary concept word, protocol supplementary concept word, rare disease supplementary concept word, unique identifier, synonyms]

2. ((Cholera or Dysentery or Diarrh*OR conjunctivitis or dengue or diphtheria or viral hepatitis, Japanese encephalitis or leishmaniosis or malaria or measles or Meningococcal meningitis or African sleeping sickness or Typhoid or Typhus or yellow fever or viral haemorrhagic fevers or Communicable disease* or Infectious disease*) adj2 (outbreak* or epidemic* or pandemic*)).mp. [mp=title, abstract, original title, name of substance word, subject heading word, floating sub-heading word, keyword heading word, organism supplementary concept word, protocol supplementary concept word, rare disease supplementary concept word, unique identifier, synonyms]

3. (Evaluation* or assessment* or appraisal* or Guidance* or Lesson*).mp. [mp=title, abstract, original title, name of substance word, subject heading word, floating sub-heading word, keyword heading word, organism supplementary concept word, protocol supplementary concept word, rare disease supplementary concept word, unique identifier, synonyms]

4. (Humanitarian adj2 (cris#s or emergenc* or disaster* or relief or aid)).mp. [mp=title, abstract, original title, name of substance word, subject heading word, floating sub-heading word, keyword heading word, organism supplementary concept word, protocol supplementary concept word, rare disease supplementary concept word, unique identifier, synonyms]

5. ((humanitarian or cris#s or emergenc* or disaster* or relief or aid or refugee* or evacuee or evacuated or displace*) adj2 (population or internal*)).mp. [mp=title, abstract, original title, name of substance word, subject heading word, floating sub-heading word, keyword heading word, organism supplementary concept word, protocol supplementary concept word, rare disease supplementary concept word, unique identifier, synonyms]

6. (war or (war adj2 (armed or zone))).mp. [mp=title, abstract, original title, name of substance word, subject heading word, floating sub-heading word, keyword heading word, organism supplementary concept word, protocol supplementary concept word, rare disease supplementary concept word, unique identifier, synonyms]

7. (conflict adj2 (armed or zone)).mp. [mp=title, abstract, original title, name of substance word, subject heading word, floating sub-heading word, keyword heading word, organism supplementary concept word, protocol supplementary concept word, rare disease supplementary concept word, unique identifier, synonyms]

8. (conflict affected adj3 (population or communit*)).mp. [mp=title, abstract, original title, name of substance word, subject heading word, floating sub-heading word, keyword heading word, organism supplementary concept word, protocol supplementary concept word, rare disease supplementary concept word, unique identifier, synonyms]

9. (earthquake or flood* or landslide* or tidal waves or tsunami* or cyclon* or typhoon* or drought or fragile state).mp. [mp=title, abstract, original title, name of substance word, subject heading word, floating sub-heading word, keyword heading word, organism supplementary concept word, protocol supplementary concept word, rare disease supplementary concept word, unique identifier, synonyms]

10. ((developing or less* developed or under developed or underdeveloped or middle income or low* income or underserved or under served or deprived or poor*) adj (economy or economies)).ti,ab.

11. ((developing or less* developed or under developed or underdeveloped or middle income or low* income or underserved or under served or deprived or poor*) adj (countr* or nation? or population? or world)).ti,ab.

12. (low* adj (gdp or gnp or gross domestic or gross national)).ti,ab.

13. (low adj3 middle adj3 countr*).ti,ab.

14. (lmic or lmics or third world or lami countr*).ti,ab.

15. transitional countr*.ti,ab.

16. global south.ti,ab.

17. Developing Countries/

18. "africa south of the sahara"/ or africa, central/ or africa, eastern/ or africa, southern/ or africa, western/

19. ("africa south of the sahara" or sub-saharan africa or central africa or eastern africa or southern africa or western africa).ti,ab.

20. "Democratic People's Republic of Korea"/

21. (north korea or (democratic people* republic adj2 korea)).ti,ab.

22. Cambodia/

23. cambodia.ti,ab.

24. Indonesia/

25. indonesia.ti,ab.

26. Micronesia/

27. Kiribati.ti,ab.

28. Laos/

29. (laos or (lao adj1 democratic republic)).ti,ab.

30. (marshall island* or caroline island* or ellice island* or gilbert island* or johnston island* or mariana island* or micronesia or pacific island*).ti,ab.

31. Mongolia/

32. mongolia.ti,ab.

33. Myanmar/

34. (myanmar or burma).ti,ab.

35. Papua New Guinea/

36. Papua New Guinea.ti,ab.

37. Philippines/

38. Philippines.ti,ab.

39. Timor-Leste/

40. Timor-Leste.ti,ab.

41. Vanuatu/

42. Vanuatu.ti,ab.

43. Vietnam/

44. (Viet Nam or Vietnam).ti,ab.

45. American Samoa/

46. american samoa.ti,ab.

47. exp China/

48. china.ti,ab.

49. Fiji/

50. fiji.ti,ab.

51. Malaysia/

52. malaysia.ti,ab.

53. marshall islands.ti,ab.

54. nauru.ti,ab.

55. samoa/

56. "independent state of samoa"/

57. ("independent state of samoa" or (samoa not american samoa) or western samoa or navigator islands or samoan islands).ti,ab.

58. Thailand/

59. Thailand.ti,ab.

60. Tonga/

61. tonga.ti,ab.

62. Tuvalu.ti,ab.

63. Armenia/

64. Armenia.ti,ab.

65. "Georgia (Republic)"/

66. Kosovo/

67. Kosovo.ti,ab.

68. Kyrgyzstan/

69. (kyrgyzstan or kyrgyz republic or kirghizia or kirghiz).ti,ab.

70. Moldova/

71. Moldova.ti,ab.

72. Tajikistan/

73. tajikistan.ti,ab.

74. Ukraine/

75. Ukraine.ti,ab.

76. Uzbekistan/

77. Uzbekistan.ti,ab.

78. Albania/

79. Albania.ti,ab.

80. Azerbaijan/

81. Azerbaijan.ti,ab.

82. "Republic of Belarus"/

83. (belarus or byelarus or belorussia).ti,ab.

84. Bosnia-Herzegovina/

85. (bosnia or herzegovina).ti,ab.

86. Bulgaria/

87. Bulgaria.ti,ab.

88. Kazakhstan/

89. (Kazakhstan or kazakh).ti,ab.

90. "Macedonia (Republic)"/

91. Macedonia.ti,ab.

92. Montenegro/

93. Montenegro.ti,ab.

94. Romania/

95. Romania.ti,ab.

96. exp Russia/

97. USSR/

98. (Russia or Russian Federation or USSR or Union of Soviet Socialist Republics or Soviet Union).mp.

99. Serbia/

100. serbia.ti,ab.

101. Turkey/

102. turkey.ti,ab. not animal/

103. Turkmenistan/

104. Turkmenistan.ti,ab.

105. Yugoslavia/

106. yugoslavia.ti,ab.

107. Haiti/

108. Haiti.ti,ab.

109. Bolivia/

110. Bolivia.ti,ab.

111. El Salvador/

112. El Salvador.ti,ab.

113. Guatemala/

114. Guatemala.ti,ab.

115. Honduras/

116. Honduras.ti,ab.

117. Nicaragua/

118. Nicaragua.ti,ab.

119. Belize/

120. Belize.ti,ab.

121. Brazil/

122. Brazil.ti,ab.

123. Colombia/

124. Colombia.ti,ab.

125. Costa Rica/

126. Costa Rica.ti,ab.

127. Cuba/

128. Cuba.ti,ab.

129. Dominica/

130. Dominica.ti,ab.

131. Dominican Republic/

132. Dominican Republic.ti,ab.

133. Ecuador/

134. Ecuador.ti,ab.

135. Grenada/

136. Grenada.ti,ab.

137. Guyana/

138. Guyana.mp.

139. Jamaica/

140. Jamaica.ti,ab.

141. Mexico/

142. Mexico.ti,ab.

143. Paraguay/

144. Paraguay.mp.

145. Peru/

146. Peru.ti,ab.

147. Saint Lucia/

148. (St Lucia or Saint Lucia).ti,ab.

149. "Saint Vincent and the Grenadines"/

150. Grenadines.ti,ab.

151. Suriname/

152. Suriname.ti,ab.

153. Venezuela/

154. Venezuela.ti,ab.

155. Djibouti/

156. (Djibouti or French Somaliland).ti,ab.

157. Egypt/

158. Egypt.ti,ab.

159. Jordan/

160. Jordan.ti,ab.

161. Morocco/

162. Morocco.ti,ab.

163. Syria/

164. (Syria or Syrian Arab Republic).ti,ab.

165. Tunisia/

166. tunisia.mp.

167. Gaza.ti,ab.

168. Yemen/

169. Yemen.ti,ab.

170. Algeria/

171. Algeria.ti,ab.

172. Iran/

173. Iran.ti,ab.

174. Iraq/

175. Iraq.ti,ab.

176. Jordan/

177. Jordan.ti,ab.

178. Lebanon/

179. Lebanon.ti,ab.

180. Libya/

181. Libya.ti,ab.

182. Afghanistan/

183. Afghanistan.ti,ab.

184. Nepal/

185. Nepal.ti,ab.

186. Bangladesh/

187. Bangladesh.ti,ab.

188. Bhutan/

189. Bhutan.ti,ab.

190. exp India/

191. India.ti,ab.

192. Pakistan/

193. Pakistan.ti,ab.

194. Sri Lanka/

195. Sri Lanka.ti,ab.

196. Indian Ocean Islands/

197. Maldives.ti,ab.

198. Benin/

199. (Benin or Dahomey).ti,ab.

200. Burkina Faso/

201. (Burkina Faso or Burkina Fasso or Upper Volta).ti,ab.

202. Burundi/

203. Burundi.ti,ab.

204. Central African Republic/

205. (Central African Republic or Ubangi-Shari).ti,ab.

206. Chad/

207. Chad.ti,ab.

208. Comoros/

209. (Comoros or Comoro Islands or Mayotte or Iles Comores).ti,ab.

210. "Democratic Republic of the Congo"/

211. ((democratic republic adj2 congo) or belgian congo or zaire).ti,ab.

212. Eritrea/

213. Eritrea.ti,ab.

214. Ethiopia/

215. Ethiopia.ti,ab.

216. Gambia/

217. Gambia.ti,ab.

218. Guinea/

219. (Guinea not (New Guinea or Guinea Pig* or Guinea Fowl)).ti,ab.

220. Guinea-Bissau/

221. (Guinea-Bissau or Portuguese Guinea).ti,ab.

222. Liberia/

223. Liberia.ti,ab.

224. Madagascar/

225. (Madagascar or Malagasy Republic).ti,ab.

226. Malawi/

227. (Malawi or Nyasaland).ti,ab.

228. Mali/

229. Mali.ti,ab.

230. Mozambique/

231. (Mozambique or Mocambique or Portuguese East Africa).ti,ab.

232. Niger/

233. (Niger not (Aspergillus or Peptococcus or Schizothorax or Cruciferae or Gobius or Lasius or Agelastes or Melanosuchus or radish or Parastromateus or Orius or Apergillus or Parastromateus or Stomoxys)).ti,ab.

234. Rwanda/

235. (Rwanda or Ruanda).ti,ab.

236. Senegal/

237. senegal.ti,ab.

238. Sierra Leone/

239. Sierra Leone.mp.

240. Somalia/

241. Somalia.ti,ab.

242. South Sudan/

243. south sudan.ti,ab.

244. Tanzania/

245. (Tanzania or Tanganyika or Zanzibar).ti,ab.

246. Togo/

247. (Togo or Togolese Republic).ti,ab.

248. Uganda/

249. Uganda.ti,ab.

250. Zimbabwe/

251. (Zimbabwe or Rhodesia).ti,ab.

252. Angola/

253. angola.ti,ab.

254. Cameroon/

255. Cameroon.ti,ab.

256. Cape Verde/

257. (Cape Verde or Cabo Verde).ti,ab.

258. Congo/

259. (congo not ((democratic republic adj3 congo) or congo red or crimean-congo)).ti,ab.

260. Cote d'Ivoire/

261. (Cote d'Ivoire or Ivory Coast).ti,ab.

262. Ghana/

263. (Ghana or Gold Coast).ti,ab.

264. Kenya/

265. kenya.mp.

266. Lesotho/

267. (Lesotho or Basutoland).ti,ab.

268. Mauritania/

269. Mauritania.ti,ab.

270. Nigeria/

271. Nigeria.ti,ab.

272. Atlantic Islands/

273. (sao tome adj2 principe).ti,ab.

274. Sudan/

275. (Sudan not south sudan).ti,ab.

276. Swaziland/

277. Swaziland.ti,ab.

278. Zambia/

279. (Zambia or Northern Rhodesia).ti,ab.

280. Botswana/

281. (Botswana or Bechuanaland or Kalahari).ti,ab.

282. Equatorial Guinea/

283. (Equatorial Guinea or Spanish Guinea).ti,ab.

284. Gabon/

285. (Gabon or Gabonese Republic).ti,ab.

286. Mauritius/

287. (Mauritius or Agalega Islands).ti,ab.

288. Namibia/

289. Namibia.ti,ab.

290. South Africa/

291. South Africa.ti,ab.

292. or/10-291 [ALL COUNTRIES DESIGNATED AS LMIC]

293. 1 or 2

294. 4 or 5 or 6 or 7 or 8 or 9

295. 292 or 294

296. 3 and 293 and 295

297. limit 296 to (yr="2010 -Current" and (english or french))

### Search Strategy CINAHL

**Interface** - EBSCOhost Research Databases
**Search Screen** - Advanced Search
**Database** - Africa-Wide Information;CINAHL Plus with Full Text;GreenFILE;Library, Information Science & Technology Abstracts

1. (outbreak* or epidemic* or pandemic*)
2. ((Cholera or Dysentery or Diarrh* or conjunctivitis or dengue or diphtheria or viral hepatitis, Japanese encephalitis or leishmaniosis or malaria or measles or Meningococcal meningitis or African sleeping sickness or Typhoid or Typhus or yellow fever or viral haemorrhagic fevers or Communicable disease* or Infectious disease*) w2 (outbreak* or epidemic* or pandemic*))
3. (Evaluation* or assessment* or appraisal* or Guidance* or Lesson*)
4. (Humanitarian w2 (cris?s or emergenc* or disaster* or relief or aid))
5. ((humanitarian or cris#s or emergenc* or disaster* or relief or aid or refugee* or evacuee or evacuated or displace*) w2 (population or internal*)).
6. (war or (war w2 (armed or zone))).
7. (conflict w2 (armed or zone))
8. (earthquake or flood* or landslide* or tidal waves or tsunami* or cyclon* or typhoon* or drought or fragile state)
9. ((developing or less* developed or under developed or underdeveloped or middle income or low* income or underserved or underserved or deprived or poor*)
10. 1 OR 2
11. 4 OR 5 OR 6 OR 7 OR 8
12. 9 OR 11
13. 3 AND 10 AND 12

### Search Strategy Web of Science

*Indexes=SCI-EXPANDED, SSCI, A&HCI, CPCI-S, CPCI-SSH, ESCI Timespan=2010-2019*

1. TS=(outbreak* OR epidemic* OR pandemic*)
2. TS=((Cholera or Dysentery or Diarrh*OR conjunctivitis or dengue or diphtheria or viral hepatitis, Japanese encephalitis or leishmaniosis or malaria or measles or Meningococcal meningitis or African sleeping sickness or Typhoid or Typhus or yellow fever or viral haemorrhagic fevers or Communicable disease* or Infectious disease*))
3. TS=(Evaluation* or assessment* or appraisal* or Guidance* or Lesson*)
4. TS=(Humanitarian (cris?s or emergenc* or disaster* or relief or aid))
5. #2 OR #1
6. (#5 AND #4 AND #3) AND LANGUAGE: (English OR French)

### WPRIM Search Strategy

1. All:appraisal OR All:assessment OR All:evaluation OR MeSH:appraisal OR MeSH:assessment OR MeSH:evaluation
2. MeSH:outbreak OR All:epidemic
3. 1 & 2

### PDQ Search Strategy

(title:((outbreak* OR epidemic* OR pandemic*)) OR abstract:((outbreak* OR epidemic* OR pandemic*))) AND (title:((Evaluation* OR assessment* OR appraisal* OR Guidance* OR Lesson)) OR abstract:((Evaluation* OR assessment* OR appraisal* OR Guidance* OR Lesson)))

1. (title:((outbreak* OR epidemic* OR pandemic*))
2. abstract:((outbreak* OR epidemic* OR pandemic*)))
3. (title:((Evaluation* OR assessment* OR appraisal* OR Guidance* OR Lesson))
4. abstract:((Evaluation* OR assessment* OR appraisal* OR Guidance* OR Lesson)))
5. 1 OR 2
6. 3 OR 4
7. 5 & 6

### Reliefweb search strategy

1. Disaster.type:epidemic
2. Theme:health
3. Format: (assessment) OR (Evaluation or Lessons Learned) OR (Manual or Guideline)
4. 1 & 2 &3
